# Mapping the global distribution of podoconiosis: Applying an evidence consensus approach

**DOI:** 10.1371/journal.pntd.0007925

**Published:** 2019-12-02

**Authors:** Kebede Deribe, Hope Simpson, Jorge Cano, David M. Pigott, Nicole Davis Weaver, Elizabeth A. Cromwell, Oliver J. Brady, Rachel L. Pullan, Abdisalan M. Noor, Daniel Argaw, Christopher J. L. Murray, Simon J. Brooker, Simon I. Hay, Melanie J. Newport, Gail Davey

**Affiliations:** 1 Department of Global Heath and Infection, Brighton and Sussex Medical School, Falmer, Brighton, United Kingdom; 2 School of Public Health, College of Health Sciences, Addis Ababa University, Addis Ababa, Ethiopia; 3 Department of Disease Control, London School of Hygiene & Tropical Medicine, London, United Kingdom; 4 Institute for Health Metrics and Evaluation, University of Washington, Seattle, Washington, United States America; 5 Centre for the Mathematical Modelling of Infectious Diseases, London School of Hygiene & Tropical Medicine, London, United Kingdom; 6 Department of Infectious Disease Epidemiology, London School of Hygiene & Tropical Medicine, London, United Kingdom; 7 Kenya Medical Research Institute-Wellcome Trust Collaborative Programme, Nairobi, Kenya; 8 Centre for Tropical Medicine and Global Health, Nuffield Department of Clinical Medicine, University of Oxford, Oxford, United Kingdom; 9 World Health Organization, Control of Neglected Tropical Diseases, Innovative and Intensified Disease Management, Geneva, Switzerland; 10 Bill & Melinda Gates Foundation, Seattle, Washington, United States of America; Johns Hopkins Bloomberg School of Public Health, UNITED STATES

## Abstract

**Background:**

Podoconiosis is a type of elephantiasis characterised by swelling of the lower legs. It is often confused with other causes of tropical lymphedema and its global distribution is uncertain. Here we synthesise the available information on the presence of podoconiosis to produce evidence consensus maps of its global geographical distribution.

**Methods and findings:**

We systematically searched available data on podoconiosis in SCOPUS and MEDLINE from inception, updated to 10 May, 2019, and identified observational and population-based studies reporting podoconiosis. To establish existence of podoconiosis, we used the number of cases reported in studies and prevalence data with geographical locations. We then developed an index to assess evidence quality and reliability, assigning each country an evidence consensus score. Using these summary scores, we then developed a contemporary global map of national-level podoconiosis status.

There is evidence of podoconiosis in 17 countries (12 in Africa, three in Latin America, and two in Asia) and consensus on presence in six countries (all in Africa). We have identified countries where surveillance is required to further define the presence or absence of podoconiosis. We have highlighted areas where evidence is currently insufficient or conflicting, and from which more evidence is needed.

**Conclusion:**

The global distribution of podoconiosis is not clearly known; the disease extent and limits provided here inform the best contemporary map of the distribution of podoconiosis globally from available data. These results help identify surveillance needs, direct future mapping activities, and inform prevention plans and burden estimation of podoconiosis.

## Author summary

The global distribution of podoconiosis is uncertain. With our current understanding of its distribution still incomplete, many of the countries suspected to be endemic for podoconiosis are based on expert opinion and lack published evidence of confirmed cases. In this study, we used multiple data sources and health metrics to identify countries with presence and absence of podoconiosis with appropriate uncertainties. After assembling a database of different evidence types we constructed a weighted score for each country called ‘evidence consensus scores’. We used these scores to measure the certainty of the presence and absence of podoconiosis. The maps produced help to identify evidence gaps and uncertainties in the current global distribution of podoconiosis. Countries with evidence of podoconiosis are mostly clustered in Africa, and a few in Asia and Latin America. We have also identified countries with indeterminate status on the presence and absences of podoconiosis. These countries are characterised by weak health systems and multiple co-endemic diseases causing lower leg swelling, potentially leading to misdiagnosis of podoconiosis. Given these challenges, we recommend intensified disease surveillance and active case searching be implemented in areas where evidence is lacking.

## Introduction

Podoconiosis is a neglected tropical disease (NTD) caused by long-term exposure to red clay soil [1–3]. The disease results from a complex interaction between genetics and the environment occurring over many years and resulting in progressive bilateral swelling of the legs. Podoconiosis has a significant impact on physical[4] and mental health[3], and imposes enormous social [5, 6]and economic [7]burdens, limiting development in communities that are often remote and disadvantaged. Despite these challenges, morbidity management is effective[8, 9], strategies for preventive behavioural change have been developed[10], and criteria for elimination have been defined[11].

In 2011, the World Health Organization (WHO) included podoconiosis on its list of other NTDs[12]. Current interventions include prevention through consistent use of footwear from an early age, regular foot hygiene and covering floors inside houses[13]. For those with the disease, the WHO-recommended lymphedema management consists of foot hygiene, foot care, wound care, compression, exercises and elevation, treatment of acute attacks, and use of shoes and socks to reduce further exposure to the irritant soil[8, 14, 15].

Despite increased global interest towards podoconiosis, its geographical distribution remains largely uncertain. Historically, podoconiosis has been reported in 32 countries globally, but there is uncertainty about its current distribution due to the lack of comprehensive surveillance[16]. Three countries (Cameroon, Ethiopia and Rwanda) have completed nationwide mapping of podoconiosis, which demonstrated substantial under-reporting of the disease [17–19]. Major reasons for underdiagnosis and/or under-reporting include profound stigma towards patients which reduces their willingness to seek healthcare[20, 21], widespread misconceptions about the cause of the disease, low awareness among health professionals and the affected communities[6, 20–22], lack of resources for disease management within programmes, and frequency of misdiagnoses as other diseases which cause lower leg lymphedema[23].

The misdiagnosis of podoconiosis is a particular challenge given the absence of point-of-care diagnostic tests, combined with scarce knowledge of the disease among health workers in many endemic countries. Misdiagnoses not only under-estimate the distribution and prevalence but may also over-estimate the burden of the misdiagnosed condition, with implications for population-at-risk estimates and programme monitoring. Thus, the joint lymphatic filariasis (LF) and podoconiosis mapping in Ethiopia conducted in 2013[24] reduced the estimated population at risk of LF from 30 million to 5.6 million[25, 26]. This was substantially due to misdiagnosis of podoconiosis lymphedema as LF cases, which had previously inflated the estimated population at risk of LF within Ethiopia.

Mapping the global distribution of a condition is a prerequisite for effective strategy and programme design. Estimating the global burden and population at risk of podoconiosis requires clear understanding of its geographical limits and a list of affected countries[27]. We aimed to synthesise the available data on podoconiosis occurrence and prevalence from both the grey and peer-reviewed literature and score podoconiosis presence or absence for all 195 countries globally. We used an evidence consensus approach[28, 29] (a measure of how strongly the combined evidence collection supports a podoconiosis-present or podoconiosis-absent status), combining these data with estimates of country-level surveillance capacity to delineate and quantify the strength of podoconiosis presence or absence globally for every country.

## Methods

### Data sources

Data sources included systematic peer-reviewed literature, conference proceedings and abstracts. We also used the information provided by Price’s monograph[2], which contains extensive personal observations and communications with experts on non-filarial elephantiasis in many parts of the world. Although it may not be very precise, this monograph[2] provides a first attempt to delineate the potential geographical distribution of this disease. Peer-reviewed literature was identified from searches of SCOPUS and MEDLINE databases, updated to 10^th^ May, 2019. Additional publications were identified from the reference lists of identified papers. The detailed search strategy and complete systematic review has been reported previously[16]. We updated this systematic search, which was carried out in 2018, to include data from recent publications[16, 30].

### Search strategy and selection criteria

We searched for studies that reported the epidemiology of podoconiosis. In May 2019 we searched SCOPUS and MEDLINE for all relevant studies that reported podoconiosis occurrence, prevalence, and incidence or case reports. We used the following search terms; “podoconiosis” OR “mossy foot” OR “non-filarial elephantiasis”. No time or language limits were applied. We hand-searched the reference lists of all recovered documents for additional references. Abstracts of all reports were read and full papers retrieved for those appearing to fulfil selection criteria. Publications were eligible for inclusion in the evidence consensus if they reported the presence of podoconiosis regardless of the type of diagnostic approach used to detect cases[30].

### Quantifying evidence with a weighted scoring system

**Fig 1:**
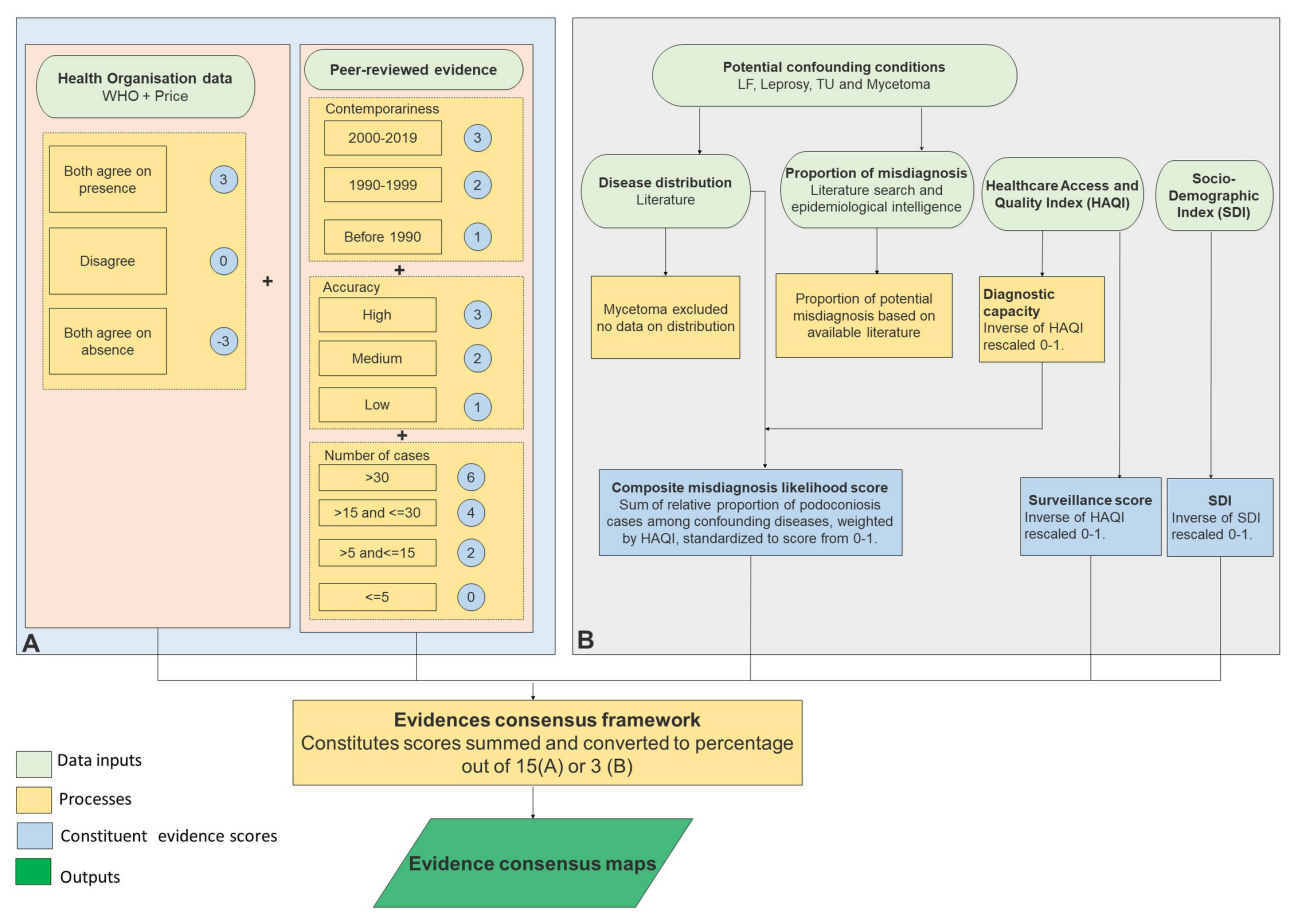
Evidence consensus framework used to assess the strength of evidence for the presence and absence of podoconiosis at the national level. A) Part A used for all countries with reported cases from any study. B) For countries with no evidence of reported cases. Maximum possible score depends on which categories are included and can vary from 15 (A) to 3 (B). HAQ = the Healthcare Access and Quality (HAQ) Index, SDI = Socio-Demographic Index.

The methodology used for generating the geographical limits of podoconiosis was adapted from previous studies on dengue[28] and the Leishmaniases[29]. Four primary evidence categories were used to determine a consensus on the presence or absence of podoconiosis: (1) health reporting organisations; (2) peer-reviewed evidence of local podoconiosis occurrence; (3) number of cases identified in surveys; (4) supplementary information assessing confounding factors known to influence podoconiosis diagnosis (Fig 1).

In order to quantify evidence consensus, a weighted scoring system termed “evidence consensus” was developed [28, 29]. This scheme attributed positive values to evidence of presence and negative values to evidence of a lack of presence. The aim was to use an optimal subset of evidence to consistently assess the strength of evidence for podoconiosis occurrence within a given area.

### Health reporting organisations

We used a map developed by WHO and a monograph written by Price[2] as a general overview for disease presence. A score of +3 was assigned if both sources agreed upon presence; 0 if they disagreed, and -3 if they agreed on absence.

### Peer-reviewed evidence

From the literature database we assessed case reports based upon the contemporariness of the data included as well as the diagnostics used to confirm podoconiosis cases. If the article contained evidence from between 2000 and 2019 a +3 score was given, while if it was between 1990 and 1999 +2 was scored and for those before 1990 +1 was given. The contemporariness of podoconiosis literature was based on the different eras of podoconiosis research. Pre-1990 studies are mostly marked based on rapid survey of people with lower leg swelling. Studies conducted between 1990 and 1999 used some form of test, whereas those studies conducted between 2000 and 2019 utilised some form of clinical algorithm including history, physical examination and disease specific tests. This has been included in the supplementary material. A variety of diagnostic methods have been described in the literature. We used the clinical algorithm developed by Sime *et al*. as a gold standard diagnosis for podoconiosis, as this is the only approach that satisfactorily eliminates other potential aetiologies. Articles using the Sime *et al*[24] approach were assigned a score of +3, those using clinical diagnosis plus immunochromatographic card test (ICT) to exclude filarial worm infection were assigned +2, and studies that simply reported cases without further diagnostic details were assigned +1.

### Number of cases reported in studies included

For each country, we added the number of cases reported in all studies included. We checked the sources of data to avoid double counting of cases. When two different studies reported cases from the same source, the original study was used to identify the case numbers. Articles/locations reporting more than 30 cases were given a score of +6, those with >15 and ≤30 were scored +4, those with >5 and ≤15 were scored +2 and those with ≤5 cases were scored 0.

### Supplementary evidence

We attempted to account for the impact of possible misdiagnoses and underreporting by assigning countries with no evidence of podoconiosis cases a score representing the evidence for absence. This score integrated three components: diagnosis, surveillance, and sociodemographic development. Each ranged from 0 to 1 and was weighted equally.

The diagnosis score was intended to reflect the likelihood of misdiagnosis of podoconiosis as other causes of lymphedema (LF, leprosy, and Tropical Ulcer (TU)), based on the distributions of these conditions and their prevalence relative to podoconiosis. We calculated the ratio of podoconiosis to each other cause of lymphedema from selected lymphedema surveys [17–19], and expressed this relationship as a proportion, reflecting a disease-specific misdiagnosis score for each disease (details in S1 Appendix). We used data on the global distributions of LF, leprosy and TU [29, 31–32] to define each country as endemic or non-endemic for each disease. For each country, the disease-specific misdiagnosis scores for its endemic diseases were summed and rescaled to 0–1, representing a composite misdiagnosis score. This score was then multiplied by a factor representing diagnostic capacity.

We measured diagnosis score and surveillance score based on the Healthcare Access and Quality (HAQ) Index values, assuming lower levels of diagnostic capacity and surveillance in countries with lower levels of personal health-care access and quality index. The HAQ Index was developed by the Global Burden of Disease (GBD) to track national healthcare access[33]. The index is a score between 0 and 100. The index was estimated by a principal component analysis of 32 causes considered amenable to health care. These causes are considered to provide a strong indication of what can and should be addressed by the recipient of effective health care, thus performance on overall personal health-care access and quality. The diagnostic capacity factor was calculated as the inverse of the HAQ Index[33] rescaled to 0–1. This factor was higher for countries with lower levels of personal health-care access and quality, giving such countries less adjustment (down-weighting) of the misdiagnosis likelihood scores, representing a higher likelihood of misdiagnosis.

The surveillance score was equal to the diagnostic capacity factor (the inverse of the HAQ Index rescaled 0–1). This score was intended to indicate low surveillance capacity, assuming lower levels of surveillance in countries with lower levels of personal health-care access and quality.

The sociodemographic index was intended to represent the propensity for podoconiosis based on socio-demographic index. The Socio-Demographic Index (SDI)[33] is a composite indicator developed by the GBD studies based on income, education, and fertility. SDI is provided in a 0–1 scale: zero represents the lowest income per capita, lowest educational attainment, and highest total fertility rate, and one represents the highest income per capita, highest educational attainment, and lowest total fertility rate[34, 35]. We used the inverse of the SDI, rescaled to 0 (most developed) to 1 (least developed) as propensity for podoconiosis.

The absence score was calculated from the sum of the diagnosis score, surveillance score and sociodemographic development score and was highest for countries co-endemic for leprosy, LF and TU, with lower levels of the HAQ Index, and lower levels of socio-demographic development[33, 35].

### Evidence consensus scoring

By scoring the evidence categories mentioned above individually and then combining their respective scores, we were able to calculate ‘‘evidence consensus,” a measure of how strongly the combined evidence collection supports a podoconiosis-present or podoconiosis-absent status. To derive an overall country evidence consensus score, the scores for all evidence categories were summed, and then divided by the maximum possible score and multiplied by 100. Consensus was defined as either consensus (± 100), very strong (±75% to ±99%), strong (±50% to ±74%), moderate (±25% to ±49%) presence or absence, indeterminate (0% to 24%), or weak evidence of absence (-1% to -24%).

The base map of the global administrative areas was downloaded from the Natural Earth (https://www.naturalearthdata.com/)[36]. All maps were produced using ArcGIS Desktop v10.5 (Environmental Systems Research Institute Inc., Redlands CA, USA).

## Results

### Global distribution of podoconiosis based on evidence consensus

**Fig 2:**
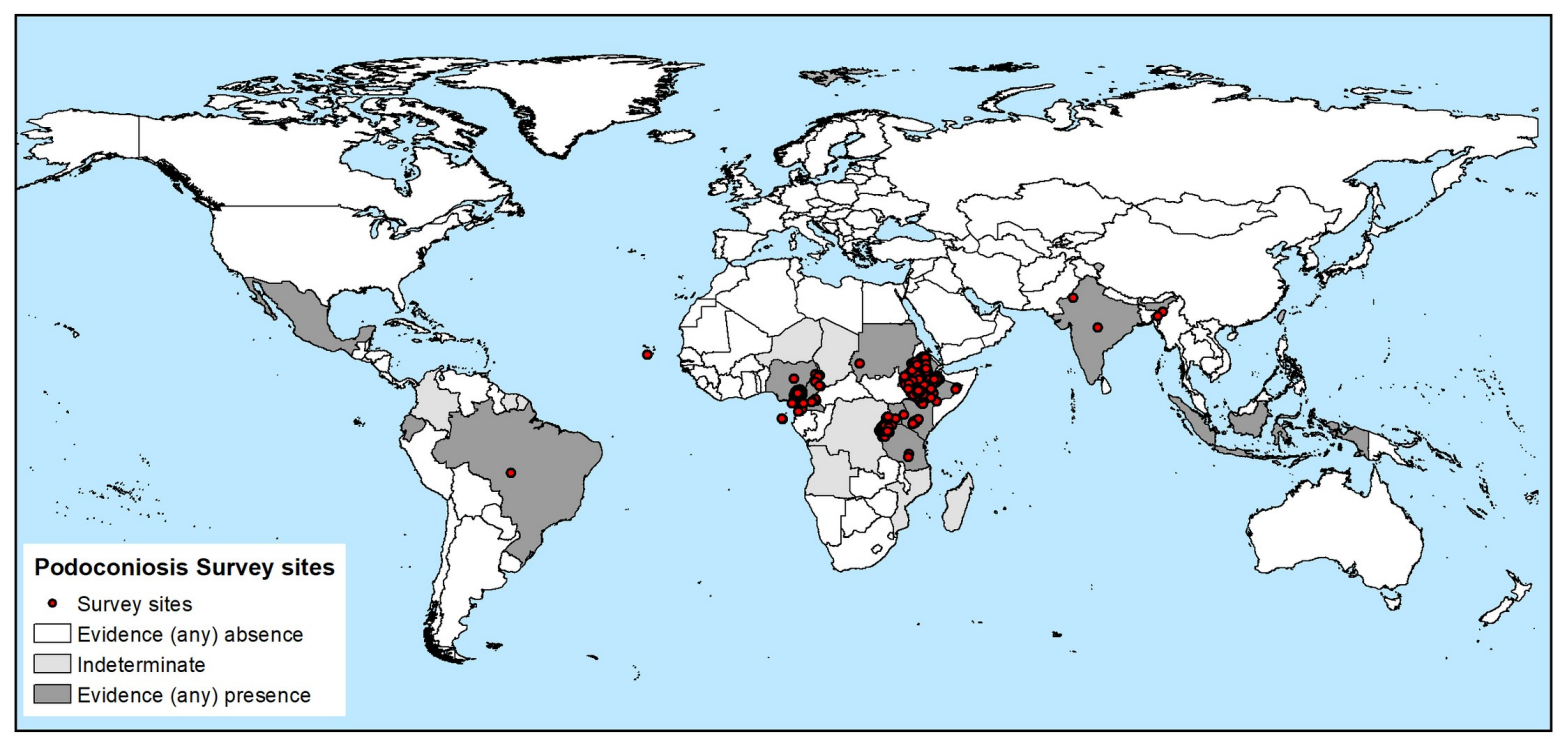
Podoconiosis occurrence data identified.

**Fig 3:**
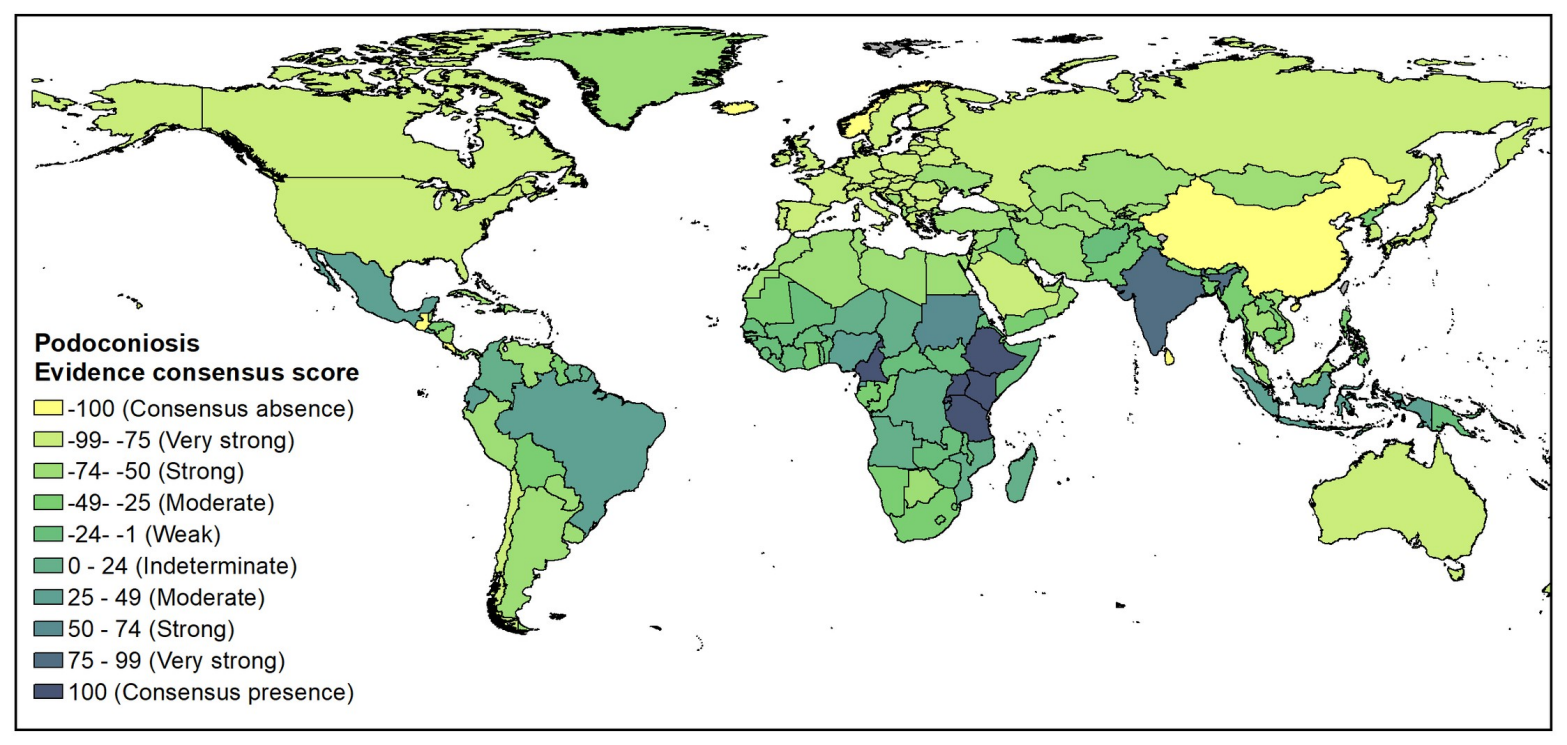
Evidence consensus for podoconiosis presence and absence worldwide. Absence of podoconiosis is yellow, areas with evidences consensus on podoconiosis status is blue.

Fig 2 shows the geographical distribution of the occurrence data included in the present study. The global distribution of podoconiosis as defined by the evidence consensus is shown in Fig 3. The mapped colour scale ranges from complete consensus on podoconiosis presence (navy), to indeterminate podoconiosis status (light green), then through to complete consensus on podoconiosis absence (yellow). The full list of the evidence used for each country and their scoring is available in Table S1. In our analysis, we identified 17 countries with evidence of podoconiosis presence (i.e. positive values outside the indeterminate range). Additionally, we have identified ten countries (Angola, Chad, Colombia, Democratic Republic of Congo, El Salvador, French Guiana, Madagascar, Mozambique, Niger and Suriname) with indeterminate status. The 17 podoconiosis-present and ten indeterminate status countries are indicated in Table 1.

**Table 1:** Evidence consensus scores for the countries with evidences of presence and indeterminate categories.

SN	Name	Score	Category
1	Cameroon	100	Consensus presence
2	Ethiopia	100	Consensus presence
3	Kenya	100	Consensus presence
4	Rwanda	100	Consensus presence
5	Uganda	100	Consensus presence
6	United Republic of Tanzania	100	Consensus presence
7	India	93	Very strong evidence for presence
8	Burundi	73	Strong evidence for presence
9	Sao Tome and Principe	67	Strong evidence for presence
10	Sudan	60	Strong evidence for presence
11	Cape Verde	60	Strong evidence for presence
12	Equatorial Guinea	47	Moderate evidence for presence
13	Nigeria	47	Moderate evidence for presence
14	Brazil	40	Moderate evidence for presence
15	Mexico	27	Moderate evidence for presence
16	Ecuador	27	Moderate evidence for presence
17	Indonesia	27	Moderate evidence for presence
18	Democratic Republic of Congo	7	Indeterminate
19	Niger	0	Indeterminate
20	Chad	0	Indeterminate
21	Madagascar	0	Indeterminate
22	Mozambique	0	Indeterminate
23	Angola	0	Indeterminate
24	El Salvador	0	Indeterminate
25	Colombia	0	Indeterminate
26	Suriname	0	Indeterminate
27	French Guiana	0	Indeterminate

### Distribution of podoconiosis in Africa

In Africa, 12 countries with podoconiosis presence were identified, with complete consensus in six (Cameroon, Ethiopia, Kenya, Tanzania, Rwanda and Uganda). The evidence in these six countries comes from local surveys and some nationwide mapping conducted in Ethiopia, Rwanda and Cameroon [17–19]. In four countries (Burundi, Cape Verde, São Tomé and Príncipe and Sudan) there was strong evidence of presence of podoconiosis [2, 37–39]. The evidence in these countries is mostly from surveys and published case reports. There was moderate evidence of presence in Equatorial Guinea and Nigeria; the evidence in Equatorial Guinea was based on a survey conducted in 1988[2, 40], whereas the evidence in Nigeria is based on a case report in 2015[41]. In addition, both WHO and Price indicated the presence of podoconiosis in Equatorial Guinea and Nigeria.

Indeterminate status was established for the Democratic Republic of Congo (DRC), although podoconiosis was reported to be present in Price’s monograph[2]. However, this was not supported by the WHO podoconiosis distribution map. The only available literature is dated to 1939 and there is no contemporary evidence on the presence of podoconiosis in the DRC[42]. In five countries (Angola, Chad, Madagascar, Mozambique and Niger) the presence of podoconiosis was reported either by WHO or Price, but there was no literature to support this, which resulted in an indeterminate status of the presence of podoconiosis in these countries.

**Fig 4:**
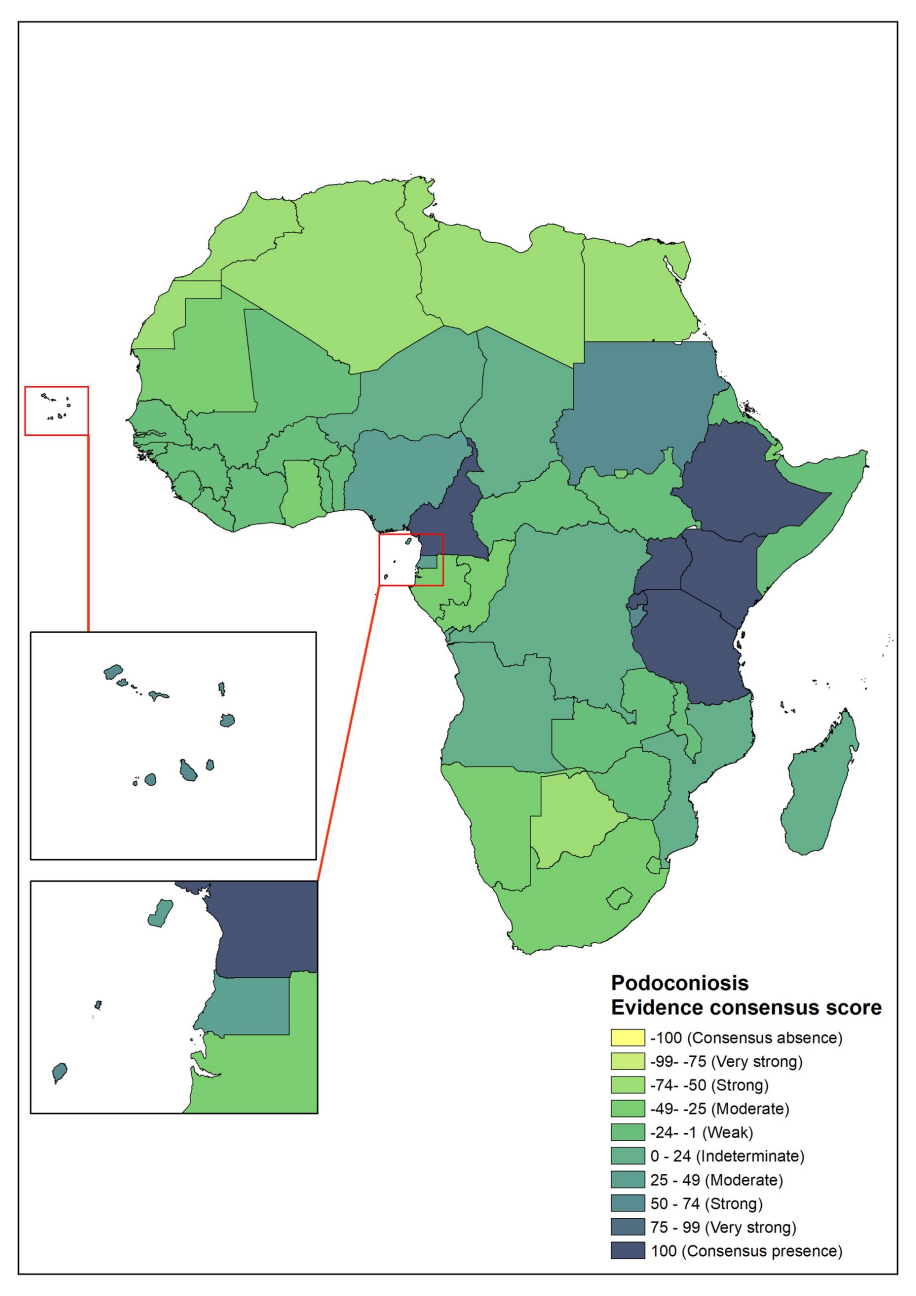
Evidence consensus for podoconiosis presence and absence in Africa. Absence of podoconiosis is yellow, areas with evidences consensus on podoconiosis status is blue.

Most parts of southern and northern Africa, including Tunisia, Libya, Egypt and Western Sahara were classified within the ‘strong evidence of absence’ category, where the disease has either never been reported or has been eliminated, as it occurs in Algeria, Morocco and Tunisia[2]. (Fig 4)

### Distribution of podoconiosis in Asia

**Fig 5:**
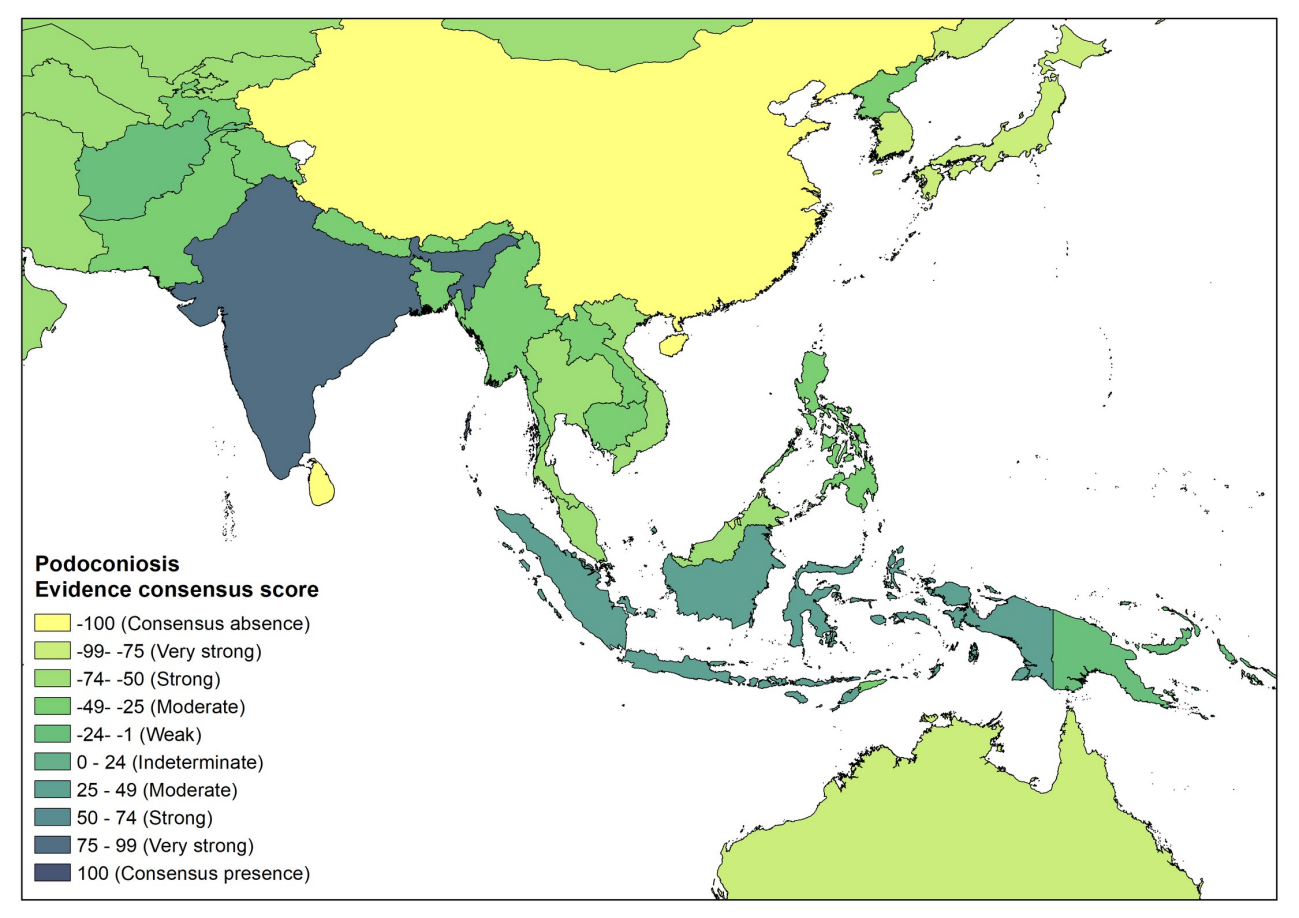
Evidence consensus for podoconiosis presence and absence in Asia. Absence of podoconiosis is yellow, areas with evidences consensus on podoconiosis status is blue.

In Asia, two countries with podoconiosis presence were identified (India and Indonesia). In India there is very strong evidence for presence, whereas in Indonesia there is moderate evidence for presence. In India, we have identified two published articles and one conference proceeding reporting on podoconiosis [43–45]. Two of the studies were conducted in cities of India (Imphal, Aizawal and Bikaner)[43, 44], whereas the third was conducted in Bhiwapur area, Nagpur district, Maharashtra[45]. In Indonesia, podoconiosis was reported in the island of Java[2]. The remaining Asian countries are characterized by absence. (Fig 5)

### Distribution of podoconiosis in Latin America

**Fig 6:**
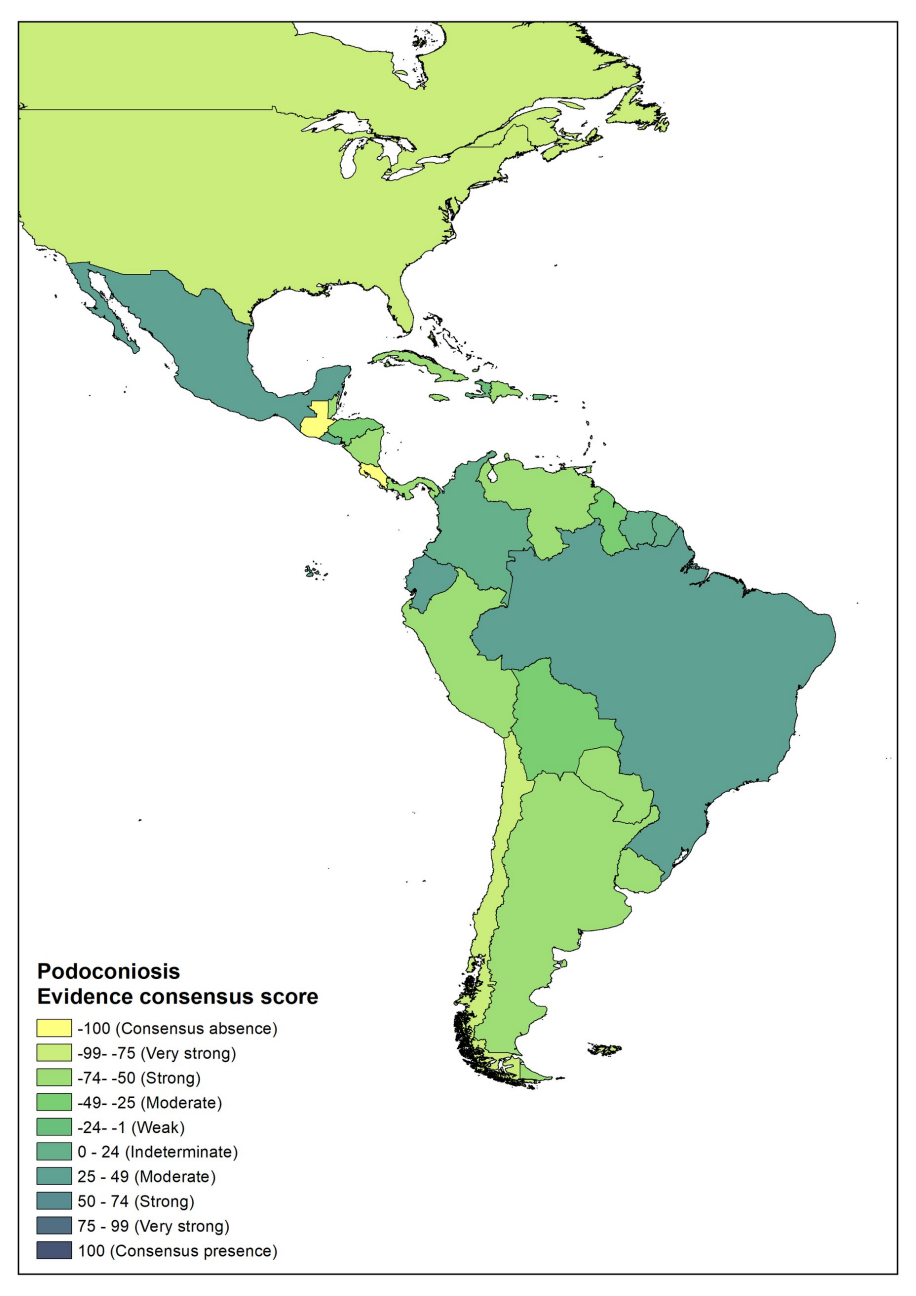
Evidence consensus for podoconiosis presence and absence in Latin America. Absence of podoconiosis is yellow, areas with evidences consensus on podoconiosis status is blue.

In Latin America there are three countries (Brazil, Ecuador and Mexico) with moderate evidence for presence of podoconiosis. In Brazil, while Price reported two cases in São Paulo[2], another study in 1993 reported a case in Central Brazil[46]. Leon, in 1952 reported the presence of podoconiosis in Ecuador, in a subtropical region about 2000 m. above sea level[47]. In addition, four countries (Colombia, El Salvador, French Guiana and Suriname) had indeterminate status, indicating that more evidence is needed (Fig 6). In all four countries, Price[2] documented the presence of podoconiosis. Nonetheless, our literature search could not identify literature on the presence of podoconiosis in these four countries.

In the rest of the world, most countries had very strong evidence or complete consensus for podoconiosis absence. There are sixteen countries with weak evidence of absence including Puerto Rico. Although neither WHO nor Price indicated the presence of podoconiosis, a publication dated 1922 mentioned the presence of podoconiosis in Puerto Rico[48]. The remaining fifteen countries are characterized by low SDI, HAQ and the presence of the three confounding diseases (LF, Leprosy and TU). Moreover, these countries neighbour countries with evidence of podoconiosis, suggesting similar environmental niches.

## Discussion

We have assembled available data on podoconiosis from multiple sources and synthesised them through an evidence consensus framework, providing the first comprehensive list of countries endemic for podoconiosis with varying levels of certainty. The maps generated provide information on the known limits of podoconiosis, and identify areas in which podoconiosis status is uncertain and requires additional information. These results have several implications for future work on podoconiosis; from estimating its global burden and limits, to targeting resources for control and case management, as well as prioritizing areas for surveillance and research.

We identified evidence of podoconiosis in 17 countries and consensus on presence in six. We have used supplementary information including the presence of confounding diseases weighted by the HAQ Index and SDI, which enabled us to identify countries with weak surveillance capacity and presence of confounding diseases which need further investigations.

Three countries with consensus on presence (Cameroon, Ethiopia and Rwanda) have already mapped the distribution of podoconiosis [17–19]. The methods applied and lessons learnt in mapping podoconiosis in these counties can be adapted to map the disease in the remaining countries, with highest priority given to those with the strongest evidence for podoconiosis. Alongside mapping activities, the implementation of morbidity management interventions should also be prioritized in countries with evidence of podoconiosis presence. Experience of mapping podoconiosis in Cameroon, Ethiopia and Rwanda indicates that the implementation of morbidity interventions in areas identified endemic will help in accelerating mapping and advocacy for elimination [24, 49]

India, Indonesia and Brazil are also among the countries with evidence of presence of podoconiosis. Given the large populations of these countries, their inclusion will have a substantial impact on the estimated population at risk globally. Taking this into account, we aim to apply an evidence consensus approach at the subnational-level in these countries, to identify whether the disease is localized or widespread throughout them. A fine-scale approach will help us to develop a robust and credible estimate of the global burden of podoconiosis and enable a tailored approach to mapping and burden estimation.

We identified ten counties with strong or moderate evidence of podoconiosis. These countries could be categorised as a second priority group in terms of mapping and evidence generation. All have historical evidence of podoconiosis presence reported in the peer-reviewed literature and Price’s monograph. In this category, Burundi is the only country in which an extensive market-based survey was conducted by Price[50]. The survey indicated widespread distribution of podoconiosis, although the data are from 1976. Most of the literature evidence from these ten countries (except Nigeria) comes from the 1980’s, which implies that there is a need to generate further evidence to qualify them. It is important that surveillance data should be collected from these countries to further inform the evidence base before deciding on nationwide mapping.

In ten countries (Angola, Chad, Colombia, Democratic Republic of Congo, El Salvador, French Guiana, Madagascar, Mozambique, Niger and Suriname), podoconiosis status was indeterminate. Neither WHO nor Price indicated the presence of podoconiosis in these countries[2], with no further peer-reviewed literature supporting this except in the Democratic Republic of Congo. It is vital to further investigate the contemporary status of podoconiosis. This can be achieved by strengthening surveillance systems to capture lymphedema cases. In addition, we plan to work with the national Ministries of Health and partners in these countries to further investigate the presence of podoconiosis through questionnaires and review of the existing health management information system[49].

The data and analysis presented herein are the first of their kind for podoconiosis. We have compiled the largest dataset available globally for podoconiosis. In addition, we have used other sources of data and evaluated potential for misclassification based on the surveillance capacities of countries and the epidemiology of confounding diseases. Hence we employed the most comprehensive approach to determine the list of countries endemic for podoconiosis. We acknowledge several limitations to our approach, however, that are important to the interpretation of the results and their future applications.

In this study, we focused on determining the presence or absence of podoconiosis at the country level. From previous work, the distribution of podoconiosis within a country has proven to be heterogeneous and we recognize the need for detailed (finer spatial resolution) information for in-country planning and intervention[51, 52]. Ongoing work with endemic countries such as India and Burundi is aimed at providing subnational administrative unit prevalence levels that are relevant to decision making for podoconiosis.

In Latin America we identified only one case report from Brazil[46], making our evidence consensus framework highly dependent on Price’s monograph[2], the country’s surveillance capacity and the presence of confounding diseases. It will be critical to generate more data from moderate evidence and indeterminate countries in the region. Our efforts to work with regional professional associations are aimed at filling these data needs.

There is a marked difference in the availability of occurrence data globally. From all the occurrence data, 79.2% were recorded from Cameroon and Ethiopia alone. This clearly indicates that there is marked variation on research on podoconiosis. Although this may not affect the current analysis, it may have implication on future analysis using the current database. Follow up analysis should account for clustering of surveys from such few countries. In addition, this implies the need for strengthening surveillance and research in other endemic countries.

The evidence consensus approach used here has several strengths such as the utilization of multiple sources of data including published literature and health metrics which measure or approximate surveillance and health system capacities. We used this method because podoconiosis is one of the most neglected tropical diseases and there is very limited understanding of its presence and absence worldwide. Integrating health system metrics into the scoring system enabled us to give an indication of the uncertainty associated with the lack of data in many countries. This approach, however, does not capture the spatial heterogeneity in the potential distribution of the disease within countries. Thus, in countries where there is high consensus of podoconiosis presence, the disease may in fact be limited to certain areas. We are addressing the problem of spatial heterogeneity in a subsequent research study, which will build upon the outcomes of present study. Finally the evidence consensus approach doesn’t provide any evidence of prevalence or burden, but could help to guide further studies to help elucidate these. The evidence consensus work presented here will be important for future work. The evidence consensus maps developed here can be integrated into a geospatial modelling framework intended to ascertain the global burden of podoconiosis. Prevalence models can be informed by the evidence consensus maps presented here.

In conclusion, we provide empirical evidence of the occurrence of podoconiosis and a list of countries endemic for the disease. Countries with the highest evidence consensus are the countries which should be targeted for further research, surveillance and initiation of prevention and control programmes. Mapping should be done in these countries to determine the in-country distribution and limits of podoconiosis which are critical for programme-level decision making[27]. Some countries with strong evidence were not historically identified as endemic for podoconiosis, which needs further study. Our compiled data form an important database for developing podoconiosis risk models and estimation of the global population at risk and ultimately burden for this avoidable NTD.

## Supporting information

S1 Appendix Evidence consensus categories results and literature sources. (DOCX)
