# Supplementary material for: Mapping the global distribution of podoconiosis: Applying an evidence consensus approach
Source: PLoS Negl Trop Dis. 2019 Dec 2;13(12):e0007925. doi: 10.1371/journal.pntd.0007925 (PMC6907864; doi:10.1371/journal.pntd.0007925)
Supplement: S1 Appendix — (DOCX) [file pntd.0007925.s001.docx]

**Supplementary Appendix 1**

**Evidence consensus categories**

1. ***Health reporting organisations***

We took maps produced by WHO and Dr Ernest Price as reference. In his monograph, Price compiled surveys conducted by himself and others, personal observation while traveling to different parts of the world and communications with experts. Endemicity classification was not done systematically; in some countries the number of cases observed was recorded while in others, presence of podoconiosis was reported without reference to literature or case detection. These data were displayed on a map showing countries potentially endemic for podoconiosis. The classification of endemicity was scored herein as follows:

**Health reporting organisations scores (-3 - +3)**

- Podoconiosis endemic by both WHO and Price: +3
- Endemic by either of the two=0
- Non-endemic by both =-3

1. ***Peer-reviewed evidence***

We searched for studies that reported the epidemiology of podoconiosis. In May 2019, we searched SCOPUS and MEDLINE for all relevant studies that examined podoconiosis occurrence, prevalence, incidence and case reports. We used the following search terms; “podoconiosis” OR “mossy foot” OR “non-filarial elephantiasis.” No time or language limits were applied. We hand-searched the reference lists of all recovered documents for additional references. Abstracts of all reports were read and full papers retrieved for those appearing to fulfil selection criteria. Publications were eligible for inclusion in the evidence consensus if they reported the presence of podoconiosis regardless of the diagnostic approach[1].

We included all literature sources and assigned scores on contemporariness, diagnostic specificity and the number of cases identified by studies. Records of cases diagnosed since 2000 were given a contemporariness score of 3. Records of cases from 1990- 1999 were given a score of 2 and those reporting prior to 1990 were given a score of 1. This classification is based on different eras of podoconiosis research.

**Contemporariness (1 -3)**

- Prior to 1990 = 1
- 1990- 1999= 2
- 2000- 2019 = 3

The diagnostic specificity score ranged from 1 to 3. The maximum score for was given for records where full exclusion of other potential causes of podoconiosis was done as described in Sime *et al*. Podoconiosis is a clinical diagnosis based on a clinical algorithm which combines history, physical examination and various disease specific tests to exclude other causes of lymphedema. Studies which used the clinical algorithm are scored +3. Those using clinical diagnosis plus immunochromatographic card test (ICT) to exclude filarial worm infection were assigned +2, and studies that simply reported cases without further diagnostic details were assigned +1.

**Diagnostic specificity (1-3):**

- Use of clinical algorithm as described in Sime et al. (2014) +3
- Clinical diagnosis plus immunochromatographic card test (ICT) or microfilarial test to exclude filarial worm infection were assigned +2,
- Only clinical diagnosis without tests and studies reported cases without further diagnostic details were assigned +1.

1. ***Number of cases reported in studies included***

For each country, we calculated the total number of cases reported in all studies included. We checked data sources to avoid double counting of cases. When two different studies reported cases from the same source, the original study was used to identify the case numbers. Countries with >30 cases scored +6, those with >15 ≤30 scored 4, those with >5 ≤15 scored 2 and those with ≤5 cases scored 0.

**Number of occurrences (0-6)**

- >30= 6
- 16-30= 4
- 6-15 = 2
- ≤5 = 0

For countries with evidence from health reporting organisations and literature evidence we used the above figures to calculate evidence consensus based on the scores. The maximum possible score in this category is 15 (all evidence categories included).

***Example***

Consensus presence (all evidence available)

- Ethiopia has been described as endemic for podoconiosis both by WHO and Price hence the Health Organisation score will be +3
- There is podoconiosis literature published in 2015 (=3) with full exclusion of other causes (=+3) therefore the country obtained +6 for the literature score.
- In a recent study, 5253 cases of podoconiosis were reported, hence the country gets +6 for number of cases reported.
- Therefore, the **evidence consensus score for Ethiopia will be** = (3+6+6)/15 *100= 100%

Indeterminate

- Niger has been described as podoconiosis endemic by WHO but Price described as non-endemic, so the Health Organisation score will be 0.
- There is no published literature on podoconiosis in Niger, so the literature sore will be 0
- No cases of podoconiosis have been reported from Niger, so the number of cases score is 0.
- Therefore, the final **evidence consensus for Niger will be** = (0+0+0)/15 *100= 0%

For the countries with no evidence of podoconiosis either by health reporting organisation or literature evidence we used supplementary evidence as follows.

1. ***Supplementary evidence***

Podoconiosis is known to occur mostly in impoverished settings, especially in populations which lack access to quality health care for diagnosis and treatment. Furthermore, the massive swelling of the lower legs and feet caused by podoconiosis is a symptom common to several other diseases, and may be misattributed to other conditions, especially in settings where there is little knowledge of podoconiosis [2-4]. We attempted to account for the impact of possible under-detection, misdiagnosis and misreporting on the strength of evidence for absence of podoconiosis, using data on the distribution of other causes of lower-limb lymphedema, estimates of the relative prevalence of podoconiosis and other conditions among lymphedema patients, an indicator of health quality and access, and an indicator of socio-demographic development.

***Estimating the likelihood of misdiagnosis***

Although podoconiosis may be misdiagnosed as multiple other infectious and non-infectious causes of lymphedema, we only included geographically restricted causes, since these would have a different effect across different countries.

We obtained data on the global distributions of lymphatic filariasis (LF), leprosy, and Tropical Ulcer (TU) from different sources [5-7]. Information on the distribution of mycetoma was not available so this disease was not included in the framework. We compiled data from surveys on the causes of lower-limb lymphedema in settings co-endemic for podoconiosis and the other conditions, providing estimates of the true proportions of podoconiosis, LF, leprosy and tropical ulcer among lymphedema patients.

We used the ratio of podoconiosis to each of the other diseases from these surveys to estimate the proportions of patients diagnosed with each other cause of lymphedema which may actually be podoconiosis cases, in countries which are co-endemic for podoconiosis but where there is limited knowledge of the disease in the health system (disease-specific misdiagnosis score). We assume that the proportions of these diseases among lymphedema patients is consistent across different settings co-endemic for the diseases.

The mapping studies conducted in Cameroon, Ethiopia, and Rwanda include estimates of the number of lymphedema cases caused by podoconiosis, LF, and leprosy, among other causes [8-10]. The retrospective cohort study in northern Ethiopia additionally estimated the proportion of cases of lymphedema resulting from TU. The overall ratio of podoconiosis to LF and leprosy was estimated from data pooled from the three surveys by Deribe et al [8-10], while the ratio of podoconiosis to TU was taken directly from the study by Padovese et al [3] (Table 1).

For each country, the disease-specific misdiagnosis scores for its endemic diseases were summed and rescaled to 0 - 1, representing a composite misdiagnosis score. This score was then multiplied by a factor representing diagnostic capacity. The diagnostic capacity factor was calculated as the inverse of the Healthcare Access and Quality (HAQ) Index [11] rescaled to 0 - 1. This factor was higher for countries with lower levels of personal health-care access and quality, giving such countries less adjustment (down-weighting) of the misdiagnosis likelihood scores, representing higher likelihood of misdiagnosis.

Healthcare Access and Quality (HAQ) Index is an index developed by the Global Burden of Diseases (GBD) to track universal health access[11]. The index is a score between 0 and 100. The index was estimated by a principal component analysis of 32 causes considered amenable to health care[12]. This causes are considered to provide strong indication of what can and should be addressed by the recipient of effective health care, thus performance on overall personal health-care access and quality. The 32 causes represent a range of health service areas including vaccine-preventable diseases; infectious diseases and maternal and child health; non-communicable diseases, including cancers, cardiovascular diseases, and other non-communicable diseases such as diabetes; and gastro intestinal conditions from which surgery can easily avert death[12]. We measured **diagnosis score** and **surveillance score** based on HAQ values, assuming lower levels of diagnostic capacity and surveillance in countries with lower levels of personal health-care access and quality index.

***Estimating the likelihood of under-detection***

The surveillance score was equal to the diagnostic capacity factor (the inverse of HQAI rescaled 0-1). This score was intended to indicate low surveillance capacity, assuming lower levels of surveillance in countries with lower levels of personal health-care access and quality.

***Estimating the propensity for podoconiosis occurrence***

The sociodemographic index was intended to represent the propensity for podoconiosis based on socio-demographic index. The Socio-Demographic Index (SDI) is a composite indicator developed by the Global Burden of Disease (GBD) studies based on income, education, and fertility [11]. SDI is scaled such that zero represents the lowest income per capita, lowest educational attainment, and highest total fertility rate, while one represents the highest income per capita, highest educational attainment, and lowest total fertility rate [13, 14]. We used the inverse of the SDI [11, 13] to indicate propensity for podoconiosis such that countries with a lower value of SDI were assigned a greater propensity for podoconiosis.

The absence score was calculated from the sum of the **diagnosis score**, **surveillance score** and **sociodemographic development score** and was highest for countries co-endemic for leprosy, LF and TU, with lower levels of HQAI, and lower levels of SDI. Maximum possible score for this category is 3.

***Example***

Philippines – Moderate evidence for absence

- There is no evidence of podoconiosis according to reporting to WHO or the Price Monograph, so the HRO score is -3.
- There is also no evidence of literature-reported cases so the framework for evidence of absence is applied.
- The Philippines are co-endemic for all three of the confounding causes of lymphedema (LF, leprosy and TU), so the composite misdiagnosis score is 1. This score is scaled down by a diagnostic factor calculated by taking the inverse of the HAQ, rescaled to 0-1. The original HAQ value is 51.8 (toward the middle of the range of values from 18.7- 97.2) so the diagnosis factor is 0.58. The adjusted composite misdiagnosis score is therefore 0.58.
- The surveillance factor is calculated in the same way to the diagnosis factor, so is 0.58.
- The original SDI is 0.62, considered middle. The SDI score is calculated as the inverse of this value; i.e. 0.41.
- This misdiagnosis, surveillance and SDI scores sum to an absence score of 1.57 out of a maximum of 3. The absence score is added to the evidence score (-3) to give an overall score of -1.43.
- This represents **-47.7% of the maximum score for absence**. This indicates that although there is no direct evidence for podoconiosis cases, the possibility of undetected or misdiagnosed cases cannot be ruled out.

Norway – Consensus absence

- There is no evidence of podoconiosis according to reporting to WHO or the Price Monograph, so the HRO score is -3.
- There is also no evidence of literature-reported cases so the framework for evidence of absence is applied.
- Norway is not co-endemic for LF, leprosy or TU, so the composite misdiagnosis score is 0.
- Norway has a high HAQ score of 96.5, the inverse of this score rescaled is 0.008. This indicates strong surveillance with a low likelihood of failure to detect incident cases.
- The original SDI of 0.91 is high, thus the calculated SDI score is low: 0.01. This represents a low propensity for podoconiosis based on the sociodemographic conditions.
- The sum of the composite misdiagnosis score, surveillance score and SDI score gives an absence score of 0.03.
- This is added to the evidence score of -3 to **give a final score of -2.97 out of 3, or -99.1%.**
- This indicates that there is no direct evidence of podoconiosis cases and strong evidence that this represents a true absence of podoconiosis.

For countries from which there is strong evidence of the absence of podoconiosis through surveillance but with no evidence of published data, the evidence score was adjusted *post-hoc*.

| **Table S1. Scores assigned based on the proportion of podoconiosis cases in a group of patients with podoconiosis and other causes** | | | | | | | | |
| --- | --- | --- | --- | --- | --- | --- | --- | --- |
| **Survey** | lymphedema | podoconiosis | LF | leprosy | TU |  |  |  |
| **Author, year** | **n** | n | n | proportion^1^ | n | proportion^2^ | n | proportion^3^ |
| **Deribe, 2015[9]** | **8,110** | 5,253 | 292 | 0.95 | 1,037 | 0.84 | NA |  |
| **Deribe, 2019[10]** | **1,143** | 914 | 38 | 0.96 | 7 | 0.99 | NA |  |
| **Padovese, 2016[3]** | **511** | 9 | 47 | 0.16 | 25 | 0.26 | 32 | 0.22 |
| **Total** | **9,847** | **6,228** | **395** | **0.94** | **1,073** | **0.85** |  | **0.22** |
| ^1^Proportion of podoconiosis cases in a group of lymphedema patients with podoconiosis or LF. ^2^Proportion of podoconiosis cases in a group of lymphedema patients with podoconiosis or leprosy. ^3^Proportion of podoconiosis cases in a group of lymphedema patients with podoconiosis or TU. | | | | | | | | |

**Table S.2:**  **Studies included in the evidence consensus and data extracted from the selected studies**

| **Country** | **Main author (year published)** | **Recording year** | **Sampling method** | **Confirmation method** | | | **Diagnosis score** | **Contemporariness score** | **Number of cases** | **Prevalence, %(95%CI)** |
| --- | --- | --- | --- | --- | --- | --- | --- | --- | --- | --- |
|  |  |  |  | **Clinical algorithm** | **Clinical diagnosis** | **No detail on diagnosis** |  |  |  |  |
| Burundi | Price, 1976[15] | 1976 | Market survey |  |  | Yes | 1 | 1 | 61 | 0.99(0.77-1.27) |
| Cape Verde | De Meira et al, 1947[16] | 1947 | Community based survey |  |  | Yes | 1 | 1 | 21 | 0.30(0.20-0.46) |
| Cameroon | Price & Henderson, 1980 [17] | 1979 | Community based survey |  |  | Yes | 1 | 1 | 130 |  |
|  | Wanji et al 2008[18] | 2006 | Community based | Yes |  |  | 3 | 3 | 66 | 7.91(6.27-9.94) |
|  | Cho-Ngwa et al., 2009[2] | 2003 | Community based |  | Yes |  | 2 | 3 | 16 | 5.32(3.30-8.46) |
|  | Wanji et al.,2016[19] | 2015 | Community based | Yes | Yes |  | 3 | 3 | 1069 | 1.90 (1.80-2.00) |
|  | Deribe et al.,2017[8] | 2017 | Community based | Yes |  |  | 3 | 3 | 52 | 0.50 (0.40-0.70) |
|  | Wanji et al.,2017[4] | 2015 | Community based |  | Yes |  | 2 | 3 | 1049 | 0.48(0.46-0.52) |
| Ethiopia | Dejene et al., 21019[20] | 2018 | Community based | Yes |  |  | 3 | 3 | 40 | 6.30(5.80-6.80) |
|  | Bekele et al.,2016[21] | 2015 | Community based |  | Yes |  | 2 | 3 | 1,197 | 3.05( 2.90-3.20) |
|  | Deribe et al, 2015[22] | 2013 | Community based | Yes |  |  | 3 | 3 | 5,253 | 4.04 (3.93–4.15) |
|  | Tekola Ayele et al, 2013[23] | 2011 | Community based |  | Yes |  | 2 | 3 | 379 | 5.65 (5.12–6.23) |
|  | Molla et al, 2012[24] | 2011 | Community based |  | Yes |  | 2 | 3 | 1,704 | 3.34 (3.19-3.50) |
|  | Geshere Oli et al, 2012[25] | 2012 | Community based | Yes |  |  | 3 | 3 | 123 | 7.43(6.26-8.79) |
|  | Alemu et al, 2011[26] | 2011 | Community based |  | Yes |  | 2 | 3 | 1,935 | 2.79(2.67–2.92) |
|  | Morrone et al 2011[27] | 2010 | Hospital based |  | Yes |  | 2 | 3 | 18 |  |
|  | Desta et al, 2007[28] | 2001 | Community based |  | Yes |  | 2 | 3 | 1890 | 5.46(5.21-5.71)* |
|  | Birrie et al., 1997[29] | 1997 | Community based |  | Yes |  | 2 | 2 | 68 | 3.58(2.83-4.51) |
|  | Formell et al, 1993[30] | 1993 | Community based |  | Yes |  | 2 | 2 | 153 | 5.06(4.33-5.90) |
|  | Kloos et al., 1992[31] | 1992 | Community based |  | Yes |  | 2 | 2 | 31 | 7.45(5.30-10.52) |
|  | Mengistu et al., 1987[32] | 1987 | Community based |  | Yes |  | 2 | 1 | 146 | 5.43(4.64-6.35) |
|  | Price,1974[33] | 1974 | Market survey |  |  | Yes | 1 | 1 | 1781 | 4.09(3.91-4.28) |
|  | Oomen, 1969[34] | 1969 | Market survey |  |  | Yes | 1 | 1 | 6770 | 2.73(2.67-2.79) |
|  | Cohen, 1960 [35] | 1954 | Facility based |  | Yes |  | 2 | 1 | 19 |  |
| Equatorial Guinea | Price 1990[16] | 1988 | Community based |  | Yes |  | 2 | 1 | 26 | 0.73 (0.50-1.07) |
|  | Corachan et al 1988[36] | 1988 | Case report | Yes |  |  | 3 | 1 | 2 |  |
| Kenya | Muli et al. 2017[37] | 2017 | Community based | Yes |  |  | 3 | 3 | 13 |  |
|  | Clark, 1948[38] | 1948 | Facility based |  | Yes |  | 2 | 1 | 200 |  |
|  | Crivelli, 1986[39] | 1986 | Community based |  | Yes |  | 2 | 1 | 105 | 3.87(3.21-4.66) |
|  | Cohen, 1960 [35] | 1958 | Facility based |  | Yes |  | 2 | 1 | 13 |  |
| Nigeria | Lar & Gusikit[40] | 2015 | Not indicated |  |  | Yes | 1 | 3 | 1 |  |
| Rwanda | Price, 1976[15] | 1976 | Market survey |  |  | Yes | 1 | 1 | 128 | 0.63(0.53-0.75) |
|  | Deribe et al 2019[10] | 2017 | Community based | Yes |  |  | 3 | 3 | 914 |  |
| Sao Tome & Principe | Ruiz 1994 [41] | 1988 | Community based | Yes |  |  | 3 | 1 | 11 | 0.92(0.52-1.64) |
| Tanzania | Eid et al.,2016[42] | 2016 | Not indicated |  |  | Yes | 1 | 3 | 1 |  |
|  | Jordan et al 1956 [43] | 1956 | Community based |  | Yes |  | 2 | 1 | 74 |  |
|  | Jordan et al., 1956 [44] | 1956 | Community based survey |  | Yes |  | 2 | 1 | 12 | 2.51(1.10-3.92) |
|  | de Lalla et al 1988 [45] | 1988 | Hospital based |  | Yes |  | 2 | 1 | 30 |  |
| Uganda | Lowenthal, 1934[46] | 1934 | Facility based |  |  | Yes | 1 | 1 | 11 |  |
|  | Onapa et al 2001[47] | 1998 | Community based |  | Yes |  | 2 | 2 | 26 | 4.52(3.10-6.54) |
|  | Dwek et al.,2015[48] | 2015 | Facility based |  | Yes |  | 2 | 3 | 3 |  |
|  | Kihembo et al., 2017[49] | 2015 | Community based | Yes |  |  | 3 | 3 | 52 |  |
| Sudan | Price, 1984[16] | 1984 | Observation |  |  | Yes | 1 | 1 | 28 |  |
|  | Price & Bailey,1884[50] | 1984 | Community based |  |  | Yes | 1 | 1 | 10 |  |
| India | Sinha M &Saha AK, 2004[51] | 2002-2003 | Facility based |  | Yes |  | 2 | 3 | 476 |  |
|  | Russel et al., 1983[52] | 1974-1982 | Community based survey |  | Yes |  | 2 | 1 | 9 | 0.21(0.11-0.40) |
|  | Kalra, 1976[53] | 1976 | Community based survey |  |  | Yes | 1 | 1 | 6 |  |
| Brazil | Tada & Marsden, 1993[54] | 1993 | Case report |  |  | Yes | 1 | 2 | 1 |  |
| Porto Rico | Ruiz-Arnau, 1922[55] | 1922 | Report |  |  | Yes | 1 | 1 | 540 |  |

**Table S.3: Evidence consensus framework results showing strength of evidence for podoconiosis presence or absence worldwide.**

Literature review scores C, D and LS show the scores assigned to the highest-scoring reference based on contemporariness (C) and diagnostic specificity (D) and combined to give a literature score (LS) score. The total cases (TC) is the sum of the total number of cases reported in all references included in the review. SDI is the Socio-Demographic Index, and HAQ the Healthcare Access and Quality Index. The Diagnostic factor was calculated as the inverse of the HAQ, SDI-I is the inverse of SDI, to indicate propensity for podoconiosis such that countries with a lower value of SDI were assigned a greater propensity for podoconiosis. Misdiagnosis scores were calculated for a range of conditions that present on lymphoedema. We used the ratio of podoconiosis to each of the other diseases from these surveys to estimate the proportions of patients diagnosed with each other cause of lymphedema which may actually be podoconiosis cases, in countries which are co-endemic for podoconiosis but where there is limited knowledge of the disease in the health system (disease-specific misdiagnosis score). The composite misdiagnosis likelihood score (CMP) is the sum of the misdiagnosis scores for all endemic diseases in each country, weighted by diagnostic factor which represents the quality of diagnosis. The evidence consensus score (ECS) positive values of ECS indicate evidence for presence, negative values indicate evidence for absence.

| NAME | Reported | | | Literature review | | | | | | | Surveillance, diagnostic capacity and propensity | | | | | Misdiagnosis likelihood score | | | | EC |
| --- | --- | --- | --- | --- | --- | --- | --- | --- | --- | --- | --- | --- | --- | --- | --- | --- | --- | --- | --- | --- |
|  | WHO | Price | HRO Sore | Literature reference | C | D | LS | TC | Refer | CS | SDI | HAQ | Propensity (SDI-I) | Diagnostic factor (HAQ-I) | Surveillance score  (HAQ-I) | LF | LPR | TU | CMP |  |
| Afghanistan | 0 | 0 | -3 | NONE | -999 |  | 0 |  |  | 0 | 0.29 | 26.42 | 0.86 | 0.90 | 0.90 | 0 | 0 | 0 | 0.0 | -11.2 |
| Albania | 0 | 0 | -3 | NONE | -999 |  | 0 |  |  | 0 | 0.68 | 75.65 | 0.32 | 0.27 | 0.27 | 0 | 0 | 0 | 0.0 | -71.0 |
| Algeria | 0 | 0 | -3 | NONE | -999 |  | 0 |  |  | 0 | 0.70 | 63.69 | 0.31 | 0.43 | 0.43 | 0 | 0 | 0.22 | 0.1 | -61.4 |
| American Samoa | 0 | 0 | -3 | NONE | -999 |  | 0 |  |  | 0 | 0.70 | 59.71 | 0.30 | 0.48 | 0.48 | 0.94 | 0 | 0 | 0.5 | -58.3 |
| Andorra | 0 | 0 | -3 | NONE | -999 |  | 0 |  |  | 0 | 0.90 | 94.72 | 0.02 | 0.03 | 0.03 | 0 | 0 | 0 | 0.0 | -97.2 |
| Angola | 0 | 1 | 0 | NONE | -999 |  | 0 |  |  | 0 | 0.46 | 34.42 | 0.63 | 0.80 | 0.80 | 0.94 | 0.85 | 0.22 | 1.0 | 0.0 |
| Antigua and Barbuda | 0 | 0 | -3 | NONE | -999 |  | 0 |  |  | 0 | 0.72 | 70.12 | 0.28 | 0.34 | 0.34 | 0 | 0 | 0 | 0.0 | -67.7 |
| Argentina | 0 | 0 | -3 | NONE | -999 |  | 0 |  |  | 0 | 0.71 | 68.49 | 0.29 | 0.37 | 0.37 | 0 | 0 | 0.22 | 0.1 | -66.1 |
| Armenia | 0 | 0 | -3 | NONE | -999 |  | 0 |  |  | 0 | 0.70 | 71.06 | 0.30 | 0.33 | 0.33 | 0 | 0 | 0 | 0.0 | -67.9 |
| Australia | 0 | 0 | -3 | NONE | -999 |  | 0 |  |  | 0 | 0.87 | 95.88 | 0.06 | 0.02 | 0.02 | 0 | 0 | 0 | 0.0 | -96.8 |
| Austria | 0 | 0 | -3 | NONE | -999 |  | 0 |  |  | 0 | 0.87 | 93.92 | 0.07 | 0.04 | 0.04 | 0 | 0 | 0 | 0.0 | -94.9 |
| Azerbaijan | 0 | 0 | -3 | NONE | -999 |  | 0 |  |  | 0 | 0.70 | 66.02 | 0.30 | 0.40 | 0.40 | 0 | 0 | 0 | 0.0 | -63.6 |
| Bahamas | 0 | 0 | -3 | NONE | -999 |  | 0 |  |  | 0 | 0.76 | 66.51 | 0.22 | 0.39 | 0.39 | 0 | 0 | 0 | 0.0 | -66.5 |
| Bahrain | 0 | 0 | -3 | NONE | -999 |  | 0 |  |  | 0 | 0.71 | 72.35 | 0.28 | 0.32 | 0.32 | 0 | 0 | 0 | 0.0 | -69.5 |
| Bangladesh | 0 | 0 | -3 | NONE | -999 |  | 0 |  |  | 0 | 0.46 | 48.35 | 0.63 | 0.62 | 0.62 | 0.94 | 0.85 | 0 | 0.9 | -37.5 |
| Barbados | 0 | 0 | -3 | NONE | -999 |  | 0 |  |  | 0 | 0.74 | 71.05 | 0.25 | 0.33 | 0.33 | 0 | 0 | 0 | 0.0 | -69.6 |
| Belarus | 0 | 0 | -3 | NONE | -999 |  | 0 |  |  | 0 | 0.77 | 79.32 | 0.20 | 0.23 | 0.23 | 0 | 0 | 0 | 0.0 | -78.2 |
| Belgium | 0 | 0 | -3 | NONE | -999 |  | 0 |  |  | 0 | 0.89 | 92.92 | 0.04 | 0.05 | 0.05 | 0 | 0 | 0 | 0.0 | -94.9 |
| Belize | 0 | 0 | -3 | NONE | -999 |  | 0 |  |  | 0 | 0.60 | 55.96 | 0.43 | 0.52 | 0.52 | 0 | 0 | 0.22 | 0.1 | -50.5 |
| Benin | 0 | 0 | -3 | NONE | -999 |  | 0 |  |  | 0 | 0.37 | 31.53 | 0.75 | 0.84 | 0.84 | 0.94 | 0 | 0.22 | 0.6 | -19.3 |
| Bermuda | 0 | 0 | -3 | NONE | -999 |  | 0 |  |  | 0 | 0.81 | 83.12 | 0.15 | 0.18 | 0.18 | 0 | 0 | 0 | 0.0 | -82.9 |
| Bhutan | 0 | 0 | -3 | NONE | -999 |  | 0 |  |  | 0 | 0.57 | 48.07 | 0.48 | 0.63 | 0.63 | 0 | 0 | 0 | 0.0 | -42.4 |
| Bolivia | 0 | 0 | -3 | NONE | -999 |  | 0 |  |  | 0 | 0.59 | 49.47 | 0.45 | 0.61 | 0.61 | 0 | 0 | 0.22 | 0.1 | -44.3 |
| Bosnia and Herzegovina | 0 | 0 | -3 | NONE | -999 |  | 0 |  |  | 0 | 0.71 | 72.55 | 0.28 | 0.31 | 0.31 | 0 | 0 | 0 | 0.0 | -69.7 |
| Botswana | 0 | 0 | -3 | NONE | -999 |  | 0 |  |  | 0 | 0.66 | 52.24 | 0.35 | 0.57 | 0.57 | 0 | 0 | 0.22 | 0.1 | -50.2 |
| Brazil | 1 | 1 | 3 | Tada & Marsden, 1993[54] | 2 | 1 | 3 | 1 | Tada & Marsden, 1993[54] | 0 | 0.66 | 64.35 | 0.35 | 0.42 | 0.42 | 0.94 | 0.85 | 0.22 | 1.0 | 40.0 |
| Brunei | 0 | 0 | -3 | NONE | -999 |  | 0 |  |  | 0 | 0.86 | 76.57 | 0.08 | 0.26 | 0.26 | 0.94 | 0 | 0 | 0.5 | -79.7 |
| Bulgaria | 0 | 0 | -3 | NONE | -999 |  | 0 |  |  | 0 | 0.79 | 77.51 | 0.17 | 0.25 | 0.25 | 0 | 0 | 0 | 0.0 | -77.5 |
| Burkina Faso | 0 | 0 | -3 | NONE | -999 |  | 0 |  |  | 0 | 0.28 | 30.70 | 0.87 | 0.85 | 0.85 | 0.94 | 0 | 0.22 | 0.6 | -14.5 |
| Burundi | 1 | 1 | 3 | Price 1976[15] | 1 | 1 | 2 | 61 | Price 1976[15] | 6 | 0.31 | 27.53 | 0.84 | 0.89 | 0.89 | 0 | 0 | 0.22 | 0.1 | 73.3 |
| Cambodia | 0 | 0 | -3 | NONE | -999 |  | 0 |  |  | 0 | 0.48 | 40.26 | 0.60 | 0.72 | 0.72 | 0.94 | 0 | 0 | 0.5 | -31.7 |
| Cameroon | 1 | 1 | 3 | Deribe et al 2017[8] | 3 | 3 | 6 | 1049 | Wanji 2018[4] | 6 | 0.48 | 32.76 | 0.60 | 0.82 | 0.82 | 0.94 | 0 | 0.22 | 0.6 | 100.0 |
| Canada | 0 | 0 | -3 | NONE | -999 |  | 0 |  |  | 0 | 0.88 | 93.83 | 0.05 | 0.04 | 0.04 | 0 | 0 | 0 | 0.0 | -95.5 |
| Cape Verde | 1 | 1 | 3 | De Meira et al, 1947[56] | 1 | 1 | 2 | 21 | De Meira et al, 1947[56] | 4 | 0.55 | 55.57 | 0.51 | 0.53 | 0.53 | 0 | 0 | 0 | 0.0 | 60.0 |
| Central African Republic | 0 | 0 | -3 | NONE | -999 |  | 0 |  |  | 0 | 0.33 | 18.66 | 0.80 | 1.00 | 1.00 | 0.94 | 0 | 0.22 | 0.6 | -6.6 |
| Chad | 1 | 0 | 0 | NONE | -999 | NA | 0 |  |  | 0 | 0.25 | 25.95 | 0.91 | 0.91 | 0.91 | 0.94 | 0 | 0.22 | 0.6 | 0.0 |
| Chile | 0 | 0 | -3 | NONE | -999 |  | 0 |  |  | 0 | 0.75 | 78.16 | 0.23 | 0.24 | 0.24 | 0 | 0 | 0.22 | 0.1 | -76.1 |
| China | 0 | 0 | -3 | NONE | -999 |  | 0 |  |  | 0 | 0.71 | 78.55 | 0.29 | 0.24 | 0.24 | 0 | 0.85 | 0 | 0.4 | -100.0 |
| Colombia | 0 | 1 | 0 | NONE | -999 | NA | 0 |  |  | 0 | 0.63 | 69.00 | 0.39 | 0.36 | 0.36 | 0 | 0 | 0.22 | 0.1 | 0.0 |
| Comoros | 0 | 0 | -3 | NONE | -999 |  | 0 |  |  | 0 | 0.43 | 33.63 | 0.66 | 0.81 | 0.81 | 0.94 | 0 | 0 | 0.5 | -23.9 |
| Congo | 0 | 0 | -3 | NONE | -999 |  | 0 |  |  | 0 | 0.57 | 34.69 | 0.47 | 0.80 | 0.80 | 0.94 | 0 | 0.22 | 0.6 | -31.2 |
| Costa Rica | 0 | 1 | 0 | NONE | -999 |  | 0 |  |  | 0 | 0.66 | 74.00 | 0.35 | 0.30 | 0.30 | 0 | 0 | 0.22 | 0.1 | -100.0 |
| Côte d'Ivoire | 0 | 0 | -3 | NONE | -999 |  | 0 |  |  | 0 | 0.41 | 28.01 | 0.70 | 0.88 | 0.88 | 0.94 | 0 | 0 | 0.5 | -18.1 |
| Croatia | 0 | 0 | -3 | NONE | -999 |  | 0 |  |  | 0 | 0.82 | 87.06 | 0.13 | 0.13 | 0.13 | 0 | 0 | 0 | 0.0 | -87.1 |
| Cuba | 0 | 0 | -3 | NONE | -999 |  | 0 |  |  | 0 | 0.69 | 76.02 | 0.32 | 0.27 | 0.27 | 0 | 0 | 0 | 0.0 | -71.5 |
| Cyprus | 0 | 0 | -3 | NONE | -999 |  | 0 |  |  | 0 | 0.86 | 90.24 | 0.07 | 0.09 | 0.09 | 0 | 0 | 0 | 0.0 | -91.7 |
| Czech Republic | 0 | 0 | -3 | NONE | -999 |  | 0 |  |  | 0 | 0.85 | 89.05 | 0.09 | 0.10 | 0.10 | 0 | 0 | 0 | 0.0 | -90.0 |
| Dem People's Rep of Korea | 0 | 0 | -3 | NONE | -999 |  | 0 |  |  | 0 | 0.54 | 53.70 | 0.52 | 0.55 | 0.55 | 0 | 0 | 0 | 0.0 | -45.7 |
| Democratic Republic of Congo | 0 | 1 | 0 | Dubois and Forro 1939 [57] | 1 |  | 1 |  |  | 0 | 0.36 | 30.58 | 0.76 | 0.85 | 0.85 | 0.94 | 0.85 | 0 | 0.9 | 6.7 |
| Denmark | 0 | 0 | -3 | NONE | -999 |  | 0 |  |  | 0 | 0.92 | 92.19 | 0.00 | 0.06 | 0.06 | 0 | 0 | 0 | 0.0 | -95.8 |
| Djibouti | 0 | 0 | -3 | NONE | -999 |  | 0 |  |  | 0 | 0.48 | 35.84 | 0.60 | 0.78 | 0.78 | 0 | 0 | 0 | 0.0 | -28.1 |
| Dominica | 0 | 0 | -3 | NONE | -999 |  | 0 |  |  | 0 | 0.69 | 62.25 | 0.32 | 0.44 | 0.44 | 0 | 0 | 0 | 0.0 | -59.7 |
| Dominican Republic | 0 | 0 | -3 | NONE | -999 |  | 0 |  |  | 0 | 0.59 | 61.73 | 0.45 | 0.45 | 0.45 | 0.94 | 0 | 0 | 0.5 | -55.0 |
| Ecuador | 1 | 1 | 3 |  | 1 |  | 1 |  |  | 0 | 0.64 | 62.66 | 0.39 | 0.44 | 0.44 | 0 | 0 | 0.22 | 0.1 | 26.7 |
| Egypt | 0 | 0 | -3 | NONE | -999 |  | 0 |  |  | 0 | 0.60 | 58.85 | 0.43 | 0.49 | 0.49 | 0.94 | 0 | 0 | 0.5 | -53.1 |
| El Salvador | 0 | 1 | 0 | NONE | -999 |  | 0 |  |  | 0 | 0.59 | 63.66 | 0.45 | 0.43 | 0.43 | 0.94 | 0 | 0.22 | 0.6 | 0.0 |
| Equatorial Guinea | 1 | 1 | 3 | Corachan et al, 1988[36] | 1 | 3 | 4 | 2 | Corachan et al, 1988[36] | 0 | 0.63 | 50.03 | 0.40 | 0.60 | 0.60 | 0.94 | 0 | 0.22 | 0.6 | 46.7 |
| Eritrea | 0 | 0 | -3 | NONE | -999 |  | 0 |  |  | 0 | 0.41 | 28.27 | 0.70 | 0.88 | 0.88 | 0.94 | 0 | 0.22 | 0.6 | -18.2 |
| Estonia | 0 | 0 | -3 | NONE | -999 |  | 0 |  |  | 0 | 0.86 | 85.95 | 0.08 | 0.14 | 0.14 | 0 | 0 | 0 | 0.0 | -87.7 |
| Ethiopia | 1 | 1 | 3 | Deribe et al 2015[9] | 3 | 3 | 6 | 5253 | Deribe et al 2015[9] | 6 | 0.33 | 29.16 | 0.80 | 0.87 | 0.87 | 0.94 | 0.85 | 0.22 | 1.0 | 100.0 |
| Fiji | 0 | 0 | -3 | NONE | -999 |  | 0 |  |  | 0 | 0.64 | 48.40 | 0.38 | 0.62 | 0.62 | 0.94 | 0 | 0 | 0.5 | -45.9 |
| Finland | 0 | 0 | -3 | NONE | -999 |  | 0 |  |  | 0 | 0.89 | 95.78 | 0.03 | 0.02 | 0.02 | 0 | 0 | 0 | 0.0 | -97.7 |
| France | 0 | 0 | -3 | NONE | -999 |  | 0 |  |  | 0 | 0.86 | 91.78 | 0.07 | 0.07 | 0.07 | 0 | 0 | 0 | 0.0 | -93.0 |
| French Guiana | 0 | 1 | 0 | NONE | -999 |  | 0 |  |  | 0 | 0.86 | 91.78 | 0.07 | 0.07 | 0.07 | 0 | 0 | 0.22 | 0.1 | 0.0 |
| Gabon | 0 | 0 | -3 | NONE | -999 |  | 0 |  |  | 0 | 0.65 | 41.16 | 0.37 | 0.71 | 0.71 | 0.94 | 0 | 0.22 | 0.6 | -40.2 |
| Gambia | 0 | 0 | -3 | NONE | -999 |  | 0 |  |  | 0 | 0.40 | 36.25 | 0.71 | 0.78 | 0.78 | 0.94 | 0 | 0.22 | 0.6 | -24.7 |
| Georgia | 0 | 0 | -3 | NONE | -999 |  | 0 |  |  | 0 | 0.85 | 67.67 | 0.10 | 0.38 | 0.38 | 0 | 0 | 0 | 0.0 | -71.8 |
| Germany | 0 | 0 | -3 | NONE | -999 |  | 0 |  |  | 0 | 0.87 | 92.02 | 0.07 | 0.07 | 0.07 | 0 | 0 | 0 | 0.0 | -93.4 |
| Ghana | 0 | 0 | -3 | NONE | -999 |  | 0 |  |  | 0 | 0.54 | 40.10 | 0.52 | 0.73 | 0.73 | 0.94 | 0 | 0.22 | 0.6 | -34.1 |
| Greece | 0 | 0 | -3 | NONE | -999 |  | 0 |  |  | 0 | 0.82 | 90.38 | 0.14 | 0.09 | 0.09 | 0 | 0 | 0 | 0.0 | -89.6 |
| Greenland | 0 | 0 | -3 | NONE | -999 |  | 0 |  |  | 0 | 0.76 | 67.72 | 0.22 | 0.38 | 0.38 | 0 | 0 | 0 | 0.0 | -67.8 |
| Grenada | 0 | 0 | -3 | NONE | -999 |  | 0 |  |  | 0 | 0.64 | 58.92 | 0.38 | 0.49 | 0.49 | 0 | 0 | 0 | 0.0 | -54.8 |
| Guatemala* | 0 | 1 | 0 | NONE | -999 |  | 0 |  |  | 0 | 0.52 | 51.93 | 0.54 | 0.58 | 0.58 | 0 | 0 | 0.22 | 0.1 | -100.0 |
| Guinea | 0 | 0 | -3 | NONE | -999 |  | 0 |  |  | 0 | 0.32 | 27.07 | 0.82 | 0.89 | 0.89 | 0.94 | 0 | 0.22 | 0.6 | -13.3 |
| Guinea-Bissau | 0 | 0 | -3 | NONE | -999 |  | 0 |  |  | 0 | 0.35 | 23.99 | 0.78 | 0.93 | 0.93 | 0.94 | 0 | 0.22 | 0.6 | -11.8 |
| Guyana | 0 | 0 | -3 | NONE | -999 |  | 0 |  |  | 0 | 0.58 | 50.35 | 0.46 | 0.60 | 0.60 | 0.94 | 0 | 0.22 | 0.6 | -44.9 |
| Haiti | 0 | 0 | -3 | NONE | -999 |  | 0 |  |  | 0 | 0.44 | 32.43 | 0.65 | 0.82 | 0.82 | 0.94 | 0 | 0 | 0.5 | -23.2 |
| Honduras | 0 | 0 | -3 | NONE | -999 |  | 0 |  |  | 0 | 0.51 | 47.04 | 0.56 | 0.64 | 0.64 | 0 | 0 | 0.22 | 0.1 | -38.8 |
| Hungary | 0 | 0 | -3 | NONE | -999 |  | 0 |  |  | 0 | 0.82 | 82.34 | 0.14 | 0.19 | 0.19 | 0 | 0 | 0 | 0.0 | -82.8 |
| Iceland | 0 | 0 | -3 | NONE | -999 |  | 0 |  |  | 0 | 0.91 | 97.18 | 0.01 | 0.00 | 0.00 | 0 | 0 | 0 | 0.0 | -99.5 |
| India | 1 | 1 | 3 | Russel et al, 1983 and Sinha & Saha Nagpur, Maharashtra 2004[51, 58] | 3 | 2 | 5 | 485 | Russel et al, 1983[58] | 6 | 0.55 | 42.19 | 0.51 | 0.70 | 0.70 | 0.94 | 0.85 | 0 | 0.9 | 93.3 |
| Indonesia | 1 | 1 | 3 | Price 1990[16] | 1 |  | 1 |  |  | 0 | 0.65 | 45.19 | 0.37 | 0.66 | 0.66 | 0.94 | 0.85 | 0.22 | 1.0 | 26.7 |
| Iran, Islamic Republic of | 0 | 0 | -3 | NONE | -999 |  | 0 |  |  | 0 | 0.70 | 72.24 | 0.30 | 0.32 | 0.32 | 0 | 0 | 0 | 0.0 | -68.8 |
| Iraq | 0 | 0 | -3 | NONE | -999 |  | 0 |  |  | 0 | 0.58 | 51.74 | 0.46 | 0.58 | 0.58 | 0 | 0 | 0 | 0.0 | -46.2 |
| Ireland | 0 | 0 | -3 | NONE | -999 |  | 0 |  |  | 0 | 0.88 | 94.62 | 0.05 | 0.03 | 0.03 | 0 | 0 | 0 | 0.0 | -96.2 |
| Israel | 0 | 0 | -3 | NONE | -999 |  | 0 |  |  | 0 | 0.82 | 84.90 | 0.14 | 0.16 | 0.16 | 0 | 0 | 0 | 0.0 | -84.9 |
| Italy | 0 | 0 | -3 | NONE | -999 |  | 0 |  |  | 0 | 0.84 | 94.88 | 0.10 | 0.03 | 0.03 | 0 | 0 | 0 | 0.0 | -94.6 |
| Jamaica | 0 | 0 | -3 | NONE | -999 |  | 0 |  |  | 0 | 0.68 | 62.22 | 0.33 | 0.45 | 0.45 | 0 | 0 | 0 | 0.0 | -59.3 |
| Japan | 0 | 0 | -3 | NONE | -999 |  | 0 |  |  | 0 | 0.87 | 94.18 | 0.07 | 0.04 | 0.04 | 0 | 0 | 0 | 0.0 | -95.0 |
| Jordan | 0 | 0 | -3 | NONE | -999 |  | 0 |  |  | 0 | 0.70 | 70.80 | 0.30 | 0.34 | 0.34 | 0 | 0 | 0 | 0.0 | -67.5 |
| Kazakhstan | 0 | 0 | -3 | NONE | -999 |  | 0 |  |  | 0 | 0.74 | 69.62 | 0.25 | 0.35 | 0.35 | 0 | 0 | 0 | 0.0 | -68.2 |
| Kenya | 1 | 1 | 3 | Muli 2017[37] | 3 | 3 | 6 | 200 | Crivelli, 1986[39] | 6 | 0.50 | 40.28 | 0.58 | 0.72 | 0.72 | 0.94 | 0 | 0.22 | 0.6 | 100.0 |
| Kiribati | 0 | 0 | -3 | NONE | -999 |  | 0 |  |  | 0 | 0.43 | 61.73 | 0.68 | 0.45 | 0.45 | 0.94 | 0 | 0 | 0.5 | -47.4 |
| Korea, Republic of | 0 | 0 | -3 | NONE | -999 |  | 0 |  |  | 0 | 0.87 | 90.48 | 0.06 | 0.09 | 0.09 | 0 | 0 | 0 | 0.0 | -92.2 |
| Kuwait | 0 | 0 | -3 | NONE | -999 |  | 0 |  |  | 0 | 0.79 | 80.97 | 0.18 | 0.21 | 0.21 | 0 | 0 | 0 | 0.0 | -80.2 |
| Kyrgyzstan | 0 | 0 | -3 | NONE | -999 |  | 0 |  |  | 0 | 0.61 | 60.99 | 0.43 | 0.46 | 0.46 | 0 | 0 | 0 | 0.0 | -55.0 |
| Lao People's Democratic Republic | 0 | 0 | -3 | NONE | -999 |  | 0 |  |  | 0 | 0.52 | 37.55 | 0.55 | 0.76 | 0.76 | 0.94 | 0 | 0 | 0.5 | -31.1 |
| Latvia | 0 | 0 | -3 | NONE | -999 |  | 0 |  |  | 0 | 0.83 | 80.88 | 0.13 | 0.21 | 0.21 | 0 | 0 | 0 | 0.0 | -81.9 |
| Lebanon | 0 | 0 | -3 | NONE | -999 |  | 0 |  |  | 0 | 0.73 | 85.86 | 0.26 | 0.14 | 0.14 | 0 | 0 | 0 | 0.0 | -81.8 |
| Lesotho | 0 | 0 | -3 | NONE | -999 |  | 0 |  |  | 0 | 0.49 | 32.62 | 0.58 | 0.82 | 0.82 | 0 | 0 | 0 | 0.0 | -25.7 |
| Liberia | 0 | 0 | -3 | NONE | -999 |  | 0 |  |  | 0 | 0.33 | 32.66 | 0.81 | 0.82 | 0.82 | 0.94 | 0 | 0.22 | 0.6 | -18.2 |
| Libyan Arab Jamahiriya | 0 | 0 | -3 | NONE | -999 |  | 0 |  |  | 0 | 0.76 | 70.75 | 0.22 | 0.34 | 0.34 | 0 | 0 | 0 | 0.0 | -70.4 |
| Lithuania | 0 | 0 | -3 | NONE | -999 |  | 0 |  |  | 0 | 0.84 | 80.60 | 0.11 | 0.21 | 0.21 | 0 | 0 | 0 | 0.0 | -82.4 |
| Luxembourg | 0 | 0 | -3 | NONE | -999 |  | 0 |  |  | 0 | 0.92 | 96.02 | 0.00 | 0.01 | 0.01 | 0 | 0 | 0 | 0.0 | -98.9 |
| Madagascar | 0 | 1 | 0 | NONE | -999 |  | 0 |  |  | 0 | 0.33 | 30.14 | 0.81 | 0.85 | 0.85 | 0.94 | 0.85 | 0.22 | 1.0 | 0.0 |
| Malawi | 0 | 0 | -3 | NONE | -999 |  | 0 |  |  | 0 | 0.35 | 32.70 | 0.78 | 0.82 | 0.82 | 0.94 | 0 | 0.22 | 0.6 | -19.2 |
| Malaysia | 0 | 0 | -3 | NONE | -999 |  | 0 |  |  | 0 | 0.76 | 68.69 | 0.22 | 0.36 | 0.36 | 0.94 | 0 | 0.22 | 0.6 | -68.5 |
| Maldives | 0 | 0 | -3 | NONE | -999 |  | 0 |  |  | 0 | 0.66 | 71.15 | 0.36 | 0.33 | 0.33 | 0.94 | 0 | 0 | 0.5 | -65.9 |
| Mali | 0 | 0 | -3 | NONE | -999 |  | 0 |  |  | 0 | 0.27 | 35.54 | 0.90 | 0.79 | 0.79 | 0.94 | 0 | 0.22 | 0.6 | -17.8 |
| Malta | 0 | 0 | -3 | NONE | -999 |  | 0 |  |  | 0 | 0.84 | 90.04 | 0.11 | 0.09 | 0.09 | 0 | 0 | 0 | 0.0 | -90.2 |
| Marshall Islands | 0 | 0 | -3 | NONE | -999 |  | 0 |  |  | 0 | 0.55 | 43.48 | 0.51 | 0.68 | 0.68 | 0.94 | 0 | 0 | 0.5 | -37.6 |
| Mauritania | 0 | 0 | -3 | NONE | -999 |  | 0 |  |  | 0 | 0.47 | 41.35 | 0.62 | 0.71 | 0.71 | 0 | 0 | 0.22 | 0.1 | -32.1 |
| Mauritius | 0 | 0 | -3 | NONE | -999 |  | 0 |  |  | 0 | 0.72 | 69.23 | 0.27 | 0.36 | 0.36 | 0 | 0 | 0 | 0.0 | -67.2 |
| Mexico | 1 | 1 | 3 | Price 1990[16] | 1 |  | 1 |  |  | 0 | 0.63 | 66.51 | 0.40 | 0.39 | 0.39 | 0 | 0 | 0 | 0.0 | 26.7 |
| Micronesia (Federated States of) | 0 | 0 | -3 | NONE | -999 |  | 0 |  |  | 0 | 0.58 | 41.98 | 0.47 | 0.70 | 0.70 | 0.94 | 0 | 0 | 0.5 | -37.4 |
| Mongolia | 0 | 0 | -3 | NONE | -999 |  | 0 |  |  | 0 | 0.66 | 53.82 | 0.35 | 0.55 | 0.55 | 0 | 0 | 0 | 0.0 | -51.4 |
| Montenegro | 0 | 0 | -3 | NONE | -999 |  | 0 |  |  | 0 | 0.79 | 81.25 | 0.18 | 0.20 | 0.20 | 0 | 0 | 0 | 0.0 | -80.5 |
| Morocco | 0 | 0 | -3 | NONE | -999 |  | 0 |  |  | 0 | 0.58 | 58.31 | 0.47 | 0.50 | 0.50 | 0 | 0 | 0 | 0.0 | -51.5 |
| Mozambique | 0 | 1 | 0 | NONE | -999 |  | 0 |  |  | 0 | 0.34 | 30.95 | 0.79 | 0.84 | 0.84 | 0.94 | 0.85 | 0.22 | 1.0 | 0.0 |
| Myanmar | 0 | 0 | -3 | NONE | -999 |  | 0 |  |  | 0 | 0.56 | 42.55 | 0.50 | 0.70 | 0.70 | 0.94 | 0.85 | 0 | 0.9 | -37.0 |
| Namibia | 0 | 0 | -3 | NONE | -999 |  | 0 |  |  | 0 | 0.62 | 45.27 | 0.42 | 0.66 | 0.66 | 0 | 0 | 0.22 | 0.1 | -42.1 |
| Nepal | 0 | 0 | -3 | NONE | -999 |  | 0 |  |  | 0 | 0.43 | 40.83 | 0.67 | 0.72 | 0.72 | 0.94 | 0.85 | 0 | 0.9 | -29.7 |
| Netherlands | 0 | 0 | -3 | NONE | -999 |  | 0 |  |  | 0 | 0.91 | 96.06 | 0.01 | 0.01 | 0.01 | 0 | 0 | 0 | 0.0 | -98.8 |
| New Zealand | 0 | 0 | -3 | NONE | -999 |  | 0 |  |  | 0 | 0.84 | 92.39 | 0.10 | 0.06 | 0.06 | 0 | 0 | 0 | 0.0 | -92.5 |
| Nicaragua | 0 | 0 | -3 | NONE | -999 |  | 0 |  |  | 0 | 0.53 | 61.80 | 0.53 | 0.45 | 0.45 | 0 | 0 | 0.22 | 0.1 | -52.2 |
| Niger | 1 | 0 | 0 | NONE | -999 |  | 0 |  |  | 0 | 0.19 | 28.83 | 1.00 | 0.87 | 0.87 | 0.94 | 0 | 0.22 | 0.6 | 0.0 |
| Nigeria | 1 | 1 | 3 | Lar & Gusikit, 2015[40] | 3 | 1 | 4 |  |  | 0 | 0.49 | 42.36 | 0.58 | 0.70 | 0.70 | 0.94 | 0.85 | 0.22 | 1.0 | 46.7 |
| Northern Mariana Islands | 0 | 0 | -3 | NONE | -999 |  | 0 |  |  | 0 | 0.76 | 73.75 | 0.22 | 0.30 | 0.30 | 0 | 0 | 0 | 0.0 | -72.8 |
| Norway | 0 | 0 | -3 | NONE | -999 |  | 0 |  |  | 0 | 0.91 | 96.54 | 0.01 | 0.01 | 0.01 | 0 | 0 | 0 | 0.0 | -99.1 |
| Oman | 0 | 0 | -3 | NONE | -999 |  | 0 |  |  | 0 | 0.74 | 76.38 | 0.24 | 0.26 | 0.26 | 0 | 0 | 0 | 0.0 | -74.3 |
| Pakistan | 0 | 0 | -3 | NONE | -999 |  | 0 |  |  | 0 | 0.49 | 38.39 | 0.59 | 0.75 | 0.75 | 0 | 0 | 0 | 0.0 | -30.6 |
| Panama | 0 | 0 | -3 | NONE | -999 |  | 0 |  |  | 0 | 0.68 | 68.98 | 0.33 | 0.36 | 0.36 | 0 | 0 | 0.22 | 0.1 | -65.0 |
| Papua New Guinea | 0 | 0 | -3 | NONE | -999 |  | 0 |  |  | 0 | 0.42 | 32.31 | 0.69 | 0.83 | 0.83 | 0.94 | 0 | 0.22 | 0.6 | -22.1 |
| Paraguay | 0 | 0 | -3 | NONE | -999 |  | 0 |  |  | 0 | 0.62 | 57.43 | 0.41 | 0.51 | 0.51 | 0 | 0 | 0 | 0.0 | -52.5 |
| Peru | 0 | 0 | -3 | NONE | -999 | NA | 0 |  |  | 0 | 0.64 | 64.85 | 0.39 | 0.41 | 0.41 | 0 | 0 | 0.22 | 0.1 | -59.6 |
| Philippines | 0 | 0 | -3 | NONE | -999 |  | 0 |  |  | 0 | 0.62 | 51.85 | 0.41 | 0.58 | 0.58 | 0.94 | 0.85 | 0.22 | 1.0 | -47.7 |
| Poland | 0 | 0 | -3 | NONE | -999 |  | 0 |  |  | 0 | 0.84 | 82.68 | 0.10 | 0.18 | 0.18 | 0 | 0 | 0 | 0.0 | -84.3 |
| Portugal | 0 | 0 | -3 | NONE | -999 |  | 0 |  |  | 0 | 0.78 | 85.89 | 0.19 | 0.14 | 0.14 | 0 | 0 | 0 | 0.0 | -84.0 |
| Puerto Rico | 0 | 0 | -3 | Ruiz -Arnau 1922[55] | 1 |  | 1 |  |  | 0 | 0.81 | 82.73 | 0.14 | 0.18 | 0.18 | 0 | 0 | 0 | 0.0 | -9.9 |
| Qatar | 0 | 0 | -3 | NONE | -999 |  | 0 |  |  | 0 | 0.77 | 81.95 | 0.21 | 0.19 | 0.19 | 0 | 0 | 0 | 0.0 | -80.1 |
| Republic of Moldova | 0 | 0 | -3 | NONE | -999 |  | 0 |  |  | 0 | 0.68 | 67.84 | 0.33 | 0.37 | 0.37 | 0 | 0 | 0 | 0.0 | -64.0 |
| Romania | 0 | 0 | -3 | NONE | -999 |  | 0 |  |  | 0 | 0.78 | 78.76 | 0.18 | 0.23 | 0.23 | 0 | 0 | 0 | 0.0 | -78.2 |
| Russian Federation | 0 | 0 | -3 | NONE | -999 |  | 0 |  |  | 0 | 0.79 | 75.38 | 0.17 | 0.28 | 0.28 | 0 | 0 | 0 | 0.0 | -75.7 |
| Rwanda | 1 | 1 | 3 | Deribe et al 2019[10] | 3 | 3 | 6 | 1143 | Deribe et al 2019[10] | 6 | 0.41 | 36.72 | 0.70 | 0.77 | 0.77 | 0 | 0 | 0.22 | 0.1 | 100.0 |
| Saint Lucia | 0 | 0 | -3 | NONE | -999 |  | 0 |  |  | 0 | 0.65 | 63.69 | 0.36 | 0.43 | 0.43 | 0 | 0 | 0 | 0.0 | -59.4 |
| Saint Vincent and the Grenadines | 0 | 0 | -3 | NONE | -999 |  | 0 |  |  | 0 | 0.61 | 57.76 | 0.43 | 0.50 | 0.50 | 0 | 0 | 0 | 0.0 | -52.3 |
| Samoa | 0 | 0 | -3 | NONE | -999 |  | 0 |  |  | 0 | 0.58 | 48.02 | 0.47 | 0.63 | 0.63 | 0.94 | 0 | 0 | 0.5 | -42.6 |
| Sao Tome and Principe | 1 | 1 | 3 | Ruiz 1994[41] | 2 | 3 | 5 | 11 | Ruiz 1994[41] | 2 | 0.49 | 40.21 | 0.59 | 0.73 | 0.73 | 0.94 | 0 | 0.22 | 0.6 | 66.7 |
| Saudi Arabia | 0 | 0 | -3 | NONE | -999 |  | 0 |  |  | 0 | 0.78 | 77.81 | 0.19 | 0.25 | 0.25 | 0 | 0 | 0 | 0.0 | -77.2 |
| Senegal | 0 | 0 | -3 | NONE | -999 |  | 0 |  |  | 0 | 0.37 | 31.64 | 0.75 | 0.83 | 0.83 | 0.94 | 0 | 0.22 | 0.6 | -19.4 |
| Serbia | 0 | 0 | -3 | NONE | -999 |  | 0 |  |  | 0 | 0.75 | 77.52 | 0.23 | 0.25 | 0.25 | 0 | 0 | 0 | 0.0 | -75.7 |
| Seychelles | 0 | 0 | -3 | NONE | -999 |  | 0 |  |  | 0 | 0.69 | 66.24 | 0.31 | 0.39 | 0.39 | 0 | 0 | 0 | 0.0 | -63.4 |
| Sierra Leone | 0 | 0 | -3 | NONE | -999 |  | 0 |  |  | 0 | 0.36 | 31.60 | 0.77 | 0.84 | 0.84 | 0.94 | 0 | 0.22 | 0.6 | -18.6 |
| Singapore | 0 | 0 | -3 | NONE | -999 |  | 0 |  |  | 0 | 0.87 | 90.73 | 0.06 | 0.08 | 0.08 | 0 | 0 | 0 | 0.0 | -92.4 |
| Slovakia | 0 | 0 | -3 | NONE | -999 |  | 0 |  |  | 0 | 0.84 | 83.49 | 0.10 | 0.17 | 0.17 | 0 | 0 | 0 | 0.0 | -84.9 |
| Slovenia | 0 | 0 | -3 | NONE | -999 |  | 0 |  |  | 0 | 0.86 | 90.86 | 0.08 | 0.08 | 0.08 | 0 | 0 | 0 | 0.0 | -92.0 |
| Solomon Islands | 0 | 0 | -3 | NONE | -999 |  | 0 |  |  | 0 | 0.43 | 32.90 | 0.68 | 0.82 | 0.82 | 0 | 0 | 0 | 0.0 | -22.8 |
| Somalia | 0 | 0 | -3 | NONE | -999 |  | 0 |  |  | 0 | 0.23 | 19.25 | 0.94 | 0.99 | 0.99 | 0 | 0 | 0.22 | 0.1 | -2.5 |
| South Africa | 0 | 0 | -3 | NONE | -999 | NA | 0 |  |  | 0 | 0.68 | 50.06 | 0.33 | 0.60 | 0.60 | 0 | 0 | 0.22 | 0.1 | -48.9 |
| South Sudan | 0 | 0 | -3 | NONE | -999 |  | 0 |  |  | 0 | 0.27 | 27.34 | 0.88 | 0.89 | 0.89 | 0.94 | 0.85 | 0 | 0.9 | -11.2 |
| Spain | 0 | 0 | -3 | NONE | -999 |  | 0 |  |  | 0 | 0.82 | 91.95 | 0.13 | 0.07 | 0.07 | 0 | 0 | 0 | 0.0 | -91.3 |
| Sri Lanka* | 1 | 1 | 3 | NONE | -999 |  | 0 |  |  | 0 | 0.68 | 71.30 | 0.33 | 0.33 | 0.33 | 0.94 | 0.85 | 0 | 0.9 | -100.0 |
| Sudan | 1 | 1 | 3 | Price & Bailey, 1984[50] | 1 | 1 | 2 | 28 | Price & Bailey, 1984[50] | 4 | 0.48 | 46.88 | 0.60 | 0.64 | 0.64 | 0.94 | 0.85 | 0.22 | 1.0 | 60.0 |
| Suriname | 0 | 1 | 0 | NONE | -999 |  | 0 |  |  | 0 | 0.64 | 54.64 | 0.38 | 0.54 | 0.54 | 0 | 0 | 0.22 | 0.1 | 0.0 |
| Swaziland | 0 | 0 | -3 | NONE | -999 |  | 0 |  |  | 0 | 0.58 | 41.01 | 0.47 | 0.72 | 0.72 | 0 | 0 | 0.22 | 0.1 | -36.7 |
| Sweden | 0 | 0 | -3 | NONE | -999 |  | 0 |  |  | 0 | 0.88 | 95.45 | 0.05 | 0.02 | 0.02 | 0 | 0 | 0 | 0.0 | -97.0 |
| Switzerland | 0 | 0 | -3 | NONE | -999 |  | 0 |  |  | 0 | 0.89 | 95.57 | 0.04 | 0.02 | 0.02 | 0 | 0 | 0 | 0.0 | -97.3 |
| Syrian Arab Republic | 0 | 0 | -3 | NONE | -999 |  | 0 |  |  | 0 | 0.61 | 67.73 | 0.42 | 0.38 | 0.38 | 0 | 0 | 0 | 0.0 | -60.9 |
| Tajikistan | 0 | 0 | -3 | NONE | -999 |  | 0 |  |  | 0 | 0.52 | 52.10 | 0.54 | 0.57 | 0.57 | 0 | 0 | 0 | 0.0 | -43.6 |
| Thailand | 0 | 0 | -3 | NONE | -999 |  | 0 |  |  | 0 | 0.68 | 70.03 | 0.32 | 0.35 | 0.35 | 0.94 | 0 | 0.22 | 0.6 | -66.2 |
| The former Yugoslav Republic of Macedonia | 0 | 0 | -3 | NONE | -999 |  | 0 |  |  | 0 | 0.75 | 75.37 | 0.22 | 0.28 | 0.28 | 0 | 0 | 0 | 0.0 | -74.0 |
| Timor-Leste | 0 | 0 | -3 | NONE | -999 |  | 0 |  |  | 0 | 0.50 | 43.66 | 0.57 | 0.68 | 0.68 | 0.94 | 0 | 0 | 0.5 | -35.6 |
| Togo | 0 | 0 | -3 | NONE | -999 |  | 0 |  |  | 0 | 0.41 | 32.78 | 0.69 | 0.82 | 0.82 | 0.94 | 0 | 0.22 | 0.6 | -22.2 |
| Tokelau | 0 | 0 | -3 | NONE | -999 |  | 0 |  |  | 0 | 0.84 | 92.39 | 0.10 | 0.06 | 0.06 | 0 | 0 | 0 | 0.0 | -92.5 |
| Tonga | 0 | 0 | -3 | NONE | -999 |  | 0 |  |  | 0 | 0.62 | 50.08 | 0.40 | 0.60 | 0.60 | 0.94 | 0 | 0 | 0.5 | -46.6 |
| Trinidad and Tobago | 0 | 0 | -3 | NONE | -999 |  | 0 |  |  | 0 | 0.70 | 64.22 | 0.30 | 0.42 | 0.42 | 0 | 0 | 0 | 0.0 | -62.0 |
| Tunisia | 0 | 0 | -3 | NONE | -999 |  | 0 |  |  | 0 | 0.68 | 69.88 | 0.33 | 0.35 | 0.35 | 0 | 0 | 0 | 0.0 | -65.7 |
| Turkey | 0 | 0 | -3 | NONE | -999 |  | 0 |  |  | 0 | 0.73 | 75.05 | 0.26 | 0.28 | 0.28 | 0 | 0 | 0 | 0.0 | -72.6 |
| Turkmenistan | 0 | 0 | -3 | NONE | -999 |  | 0 |  |  | 0 | 0.70 | 62.32 | 0.30 | 0.44 | 0.44 | 0 | 0 | 0 | 0.0 | -60.3 |
| U.K. of Great Britain and Northern Ireland | 0 | 0 | -3 | NONE | -999 |  | 0 |  |  | 0 | 0.84 | 90.58 | 0.10 | 0.08 | 0.08 | 0 | 0 | 0 | 0.0 | -91.0 |
| Uganda | 1 | 1 | 3 | Kihembo et al 2015[49] | 3 | 3 | 6 | 202 | Kihembo et al 2015[49] | 6 | 0.39 | 32.17 | 0.73 | 0.83 | 0.83 | 0.94 | 0 | 0.22 | 0.6 | 100.0 |
| Ukraine | 0 | 0 | -3 | NONE | -999 |  | 0 |  |  | 0 | 0.74 | 74.77 | 0.24 | 0.29 | 0.29 | 0 | 0 | 0 | 0.0 | -72.8 |
| United Arab Emirates | 0 | 0 | -3 | NONE | -999 |  | 0 |  |  | 0 | 0.79 | 70.45 | 0.17 | 0.34 | 0.34 | 0 | 0 | 0 | 0.0 | -71.7 |
| United Republic of Tanzania | 1 | 1 | 3 | Eid et al 2016[42] | 3 | 3 | 6 | 31 | de Lalla et al 1988[45] | 6 | 0.41 | 34.75 | 0.70 | 0.80 | 0.80 | 0.94 | 0.85 | 0 | 0.9 | 100.0 |
| United States of America | 0 | 0 | -3 | NONE | -999 |  | 0 |  |  | 0 | 0.87 | 88.78 | 0.07 | 0.11 | 0.11 | 0 | 0 | 0 | 0.0 | -90.5 |
| Uruguay | 0 | 0 | -3 | NONE | -999 |  | 0 |  |  | 0 | 0.71 | 71.55 | 0.29 | 0.33 | 0.33 | 0 | 0 | 0 | 0.0 | -68.6 |
| Uzbekistan | 0 | 0 | -3 | NONE | -999 |  | 0 |  |  | 0 | 0.63 | 63.41 | 0.40 | 0.43 | 0.43 | 0 | 0 | 0 | 0.0 | -58.1 |
| Vanuatu | 0 | 0 | -3 | NONE | -999 |  | 0 |  |  | 0 | 0.48 | 32.77 | 0.61 | 0.82 | 0.82 | 0.94 | 0 | 0 | 0.5 | -25.0 |
| Venezuela | 0 | 0 | -3 | NONE | -999 |  | 0 |  |  | 0 | 0.66 | 67.95 | 0.36 | 0.37 | 0.37 | 0 | 0 | 0.22 | 0.1 | -63.1 |
| Viet Nam | 0 | 0 | -3 | NONE | -999 |  | 0 |  |  | 0 | 0.61 | 61.08 | 0.43 | 0.46 | 0.46 | 0.94 | 0 | 0 | 0.5 | -55.1 |
| Virgin Islands | 0 | 0 | -3 | NONE | -999 |  | 0 |  |  | 0 | 0.81 | 74.05 | 0.00 |  | 1.00 | 0 | 0 | 0 | 0.0 | -66.7 |
| Western Sahara | 0 | 0 | -3 | NONE | -999 |  | 0 |  |  | 0 | 0.58 | 58.31 | 0.47 | 0.50 | 0.50 | 0 | 0 | 0 | 0.0 | -51.5 |
| Yemen | 0 | 0 | -3 | NONE | -999 |  | 0 |  |  | 0 | 0.43 | 43.37 | 0.67 | 0.69 | 0.69 | 0.94 | 0 | 0 | 0.5 | -31.9 |
| Zambia | 0 | 0 | -3 | NONE | -999 |  | 0 |  |  | 0 | 0.47 | 29.72 | 0.61 | 0.86 | 0.86 | 0.94 | 0 | 0.22 | 0.6 | -22.3 |
| Zimbabwe | 0 | 0 | -3 | NONE | -999 |  | 0 |  |  | 0 | 0.46 | 31.88 | 0.63 | 0.83 | 0.83 | 0.94 | 0 | 0.22 | 0.6 | -23.7 |
| C=contemporariness score; D=diagnosis score; TC=total number of cases; CS=case number score. LPR=leprosy, LF=lymphatic filariasis, TU=tropical ulcer, CMP=composite. ECS= evidence consensus score. SDI=Socio-Demographic Index, and HAQI=Healthcare Access and Quality Index, *Score adjusted *post hoc*. | | | | | | | | | | | | | | | | | | | | |

**References**

1. Deribe K, Cano K, Davey G. Global epidemiology of podoconiosis: a systematic review . PROSPERO: CRD42018084959 Available from: <http://www.crd.york.ac.uk/PROSPERO/display_record.php?ID=CRD42018084959>. 2018.

2. Cho-Ngwa F, Amambua AN, Ambele MA, Titanji VPK. Evidence for the exacerbation of lymphedema of geochemical origin, podoconiosis, by onchocerciasis. Journal of Infection and Public Health 2009;2:198-203.

3. Padovese V, Marrone R, Dassoni F, Vignally P, Barnabas GA, Morrone A. The diagnostic challenge of mapping elephantiasis in the Tigray region of northern Ethiopia. Int J Dermatol 2016;55(5):563-770.

4. Wanji S, Kengne-Ouafo JA, Deribe K, Tembei AM, Jelil AN, Bita DT, et al. Study of Lymphoedema of Non-Filarial Origin in the North West Region of Cameroon: Spatial Distribution, Profiling of Cases and Socio-economic Aspects of Podoconiosis Int Health. 2017;10(4):285-93.

5. Berger S. Tropical Skin Ulcers: Global Status. Gideon Informatics Inc., editor2018.

6. Pigott DM, Bhatt S, Golding N, Duda KA, Battle KE, Brady OJ, et al. Global Distribution Maps of the Leishmaniases. Elife. 2014;e02851.

7. Pigott DM, Bhatt S, Golding N, Duda KA, Battle KE, Brady OJ, et al. Data from: Global distribution maps of the Leishmaniases. Compiled: Dryad Data Repository. 2014.

8. Deribe K, Andrew AB, Cano J, Jelil A, Fru-Cho J, Raphael A, et al. Mapping the geographical distribution of podoconiosis in Cameroon using parasitological, serological, and clinical evidence to exclude other causes of lymphedema. PLoS Negl Trop Dis. 2017 12(1):e0006126.

9. Deribe K, Brooker SJ, Pullan RL, Sime H, Gebretsadik A, Assefa A, et al. Epidemiology and individual, household and geographical risk factors of podoconiosis in Ethiopia: results from the first nationwide mapping. Am J Trop Med Hyg. 2015;92(1):148–58.

10. Deribe K, Mbituyumuremyi A, Cano J, Jean Bosco M, Giorgi E, Ruberanziza E, et al. Geographical distribution and prevalence of podoconiosis in Rwanda: a cross-sectional country-wide survey. Lancet Glob Health. 7(5):e671-e80.

11. Global Burden of Disease Collaborative Network. Global Burden of Disease Study 2017 (GBD 2017) Covariates 1980-2017. Seattle, United States: Institute for Health Metrics and Evaluation (IHME). . 2018.

12. GBD 2016 Healthcare Access and Quality Collaborators. Measuring performance on the Healthcare Access and Quality Index for 195 countries and territories and selected subnational locations: a systematic analysis from the Global Burden of Disease Study 2016. Lancet 2018;391:2236-.

13. Global Burden of Disease Collaborative Network. Global Burden of Disease Study 2015 (GBD 2015) Socio-Demographic Index (SDI) 1980–2015. Seattle, United States: Institute for Health Metrics and Evaluation (IHME), 2016. 2016.

14. GBD 2017 Risk Factor Collaborators. Global, regional, and national comparative risk assessment of 84 behavioural, environmental and occupational, and metabolic risks or clusters of risks for 195 countries and territories, 1990-2017: a systematic analysis for the Global Burden of Disease Study 2017. Lancet. 2018;392(10159):1923-94.

15. Price EW. Endemic elephantiasis of the lower legs in Rwanda and Burundi. Trop Geogr Med. 1976;28 283-90.

16. Price E. Podoconiosis:Non-filarial Elephantiasis. Oxford Medical Publications, Oxford, UK. 1990.

17. Price EW, Henderson WJ. Endemic elephantiasis of the lower legs in the United Cameroon Republic. Trop Geogr Med 1981;33(1):23-9.

18. Wanji S, Tendongfor N, Esum M, Che JN, Mand S, Tanga Mbi C, et al. Elephantiasis of non-filarial origin (podoconiosis) in the highlands of north-western Cameroon. Ann Trop Med Parasitol. 2008;102(6):529-40.

19. Wanji S, Kengne-Ouafo JA, Datchoua-Poutcheu FR, Njouendou AJ, Tayong DB, Sofeu-Feugaing DD, et al. Detecting and staging podoconiosis cases in North West Cameroon: positive predictive value of clinical screening of patients by community health workers and researchers. BMC Public Health. 2016;16:997.

20. Dejene F, Merga H, Asefa H. Community based cross sectional study of podoconiosis and associated factors in Dano district, Central Ethiopia. PLoS Negl Trop Dis. 2019;13(1):e0007050.

21. Bekele K, Samuel A, Ambebir T, Deribe K, Davey G. Burden of Podoconiosis in Wayu Tuka Woreda, East Wollega Zone, Western Ethiopia. BMJ Open. 2016;6(9):e012308.

22. Deribe K, Brooker SJ, Pullan RL, Sime H, Gebretsadik A, Assefa A, et al. Epidemiology and individual, household and geographical risk factors of podoconiosis in Ethiopia: results from the first nationwide mapping. Am J Trop Med Hyg. 2015:148–58.

23. Tekola Ayele F, Alemu G, Davey G, Ahrens C. Community-based survey of podoconiosis in Bedele Zuria woreda, southwest Ethiopia. Int Health. 2013; 5(2):119-25.

24. Molla YB, Tomczyk S, Amberbir T, Tamiru A, Davey G. Podoconiosis in East and West Gojam zones, Northern Ethiopia. PLoS Negl Trop Dis. 2012;6(7):e1744.

25. Geshere Oli G, Tekola Ayele F, Petros B. Parasitological, serological, and clinical evidence for high prevalence of podoconiosis (non-filarial elephantiasis) in Midakegn district, central Ethiopia. Trop Med Int Health. 2012;17(6):722-6. doi: 10.1111/j.1365-3156.2012.02978.

26. Alemu G, Tekola Ayele F, Daniel T, Ahrens C, Davey G. Burden of podoconiosis in poor rural communities in Gulliso woreda, West Ethiopia. PLoS Negl Trop Dis. 2011;5(6):e1184.

27. Morrone A, Padovese V, Dassoni F, Pajno MC, Marrone R, Franco G, et al. Podoconiosis: an experience from Tigray, Northern Ethiopia. J Am Acad Dermatol. 2011;65(1):214-5.

28. Desta K, Ashine M, Davey G. Prevalence of podoconiosis (endemic non-filarial elephantiasis) in Wolaitta, Southern Ethiopia Tropical Doctor. 2003;32:217-20.

29. Birrie H, Balcha F, Jemaneh L. Elephantiasis in Pawe settlement area: podoconiosis or Bancroftian filariasis? . Ethiopian Medical Journal. 1997;35:245-50.

30. Frommel D, Ayranci B, Pfeifer HR, Sanchez A, Frommel A, Mengistu G. Podoconiosis in the Ethiopian Rift Valley. Role of beryllium and zirconium. Trop Geogr Med 1993;45(4):165-7.

31. Kloos H, Kello AB, Addus A. Podoconiosis (endemic non-filarial elephantiasis) in two resettlement schemes in western Ethiopia. Tropical Doctor. 1992; 22:109-12.

32. Mengistu G, Humber D, Ersumo M, Mamo T. High prevalence of elephantiasis and cutaneous leishmaniasis in Ocholo, south-west Ethiopia. . Ethiopian Medical Journal. 1987;25:203-7.

33. Price EW. The relationship between endemic elephantiasis of the lower legs and the local soils and climate : A study in Wollamo District, Southern Ethiopia. Trop Geogr Med 1974;26(3):225-30.

34. Oomen AP. Studies on elephantiasis of the legs in Ethiopia. Trop Geogr Med. 1969;1969 (21):3.

35. Cohen LB. Idiopathic lymphoedema of Ethiopia and Kenya. East Afr Med J. 1960;37:53-74.

36. Corachan M, Tura JM, Campo E, Soley M, Traveria A. Podoconiosis in Aequatorial Guinea. Report of two cases from different geological environments. Trop Geogr Med 1988;40(4):359-64.

37. Muli J, Gachohi J, Kagai J. Soil iron and aluminium concentrations and feet hygiene as possible predictors of Podoconiosis occurrence in Kenya. PLoS Negl Trop Dis. 2017;11(8):e0005864.

38. Clark M. Lymphostatic Verrucosis in the Fort Hall District of Kenya. Trans R Soc Trop Med Hyg. 1948;42(3):287-90.

39. Crivelli PE. Non-filarial elephantiasis in Nyambene range: a geochemical disease. East Afr Med J. 1986;63(3):191-4.

40. Lar UA, Gusikit RB. Environmental and health impact of potentially harmful elements distribution in the Panyam (Sura) volcanic province, Jos Plateau, Central Nigeria. Environmental Earth Sciences. 2015;74(2):1699-710.

41. Ruiz L, Campo E, Corachan M. Elephantiasis in Sao Tome and Principe. Acta Trop. 1994;57(1):29-34.

42. Eid R, Sharma D, Smock W. Images in Clinical Tropical Medicine Podoconiosis in Rural Tanzania. Am J Trop Med Hyg. 2016;95(1):1.

43. Jordan P, Hope Trant M, Laurie W. Non-Bancroftian Elephantiasis in Tanganyika. British Medical Journal 1956;28:209-10.

44. Jordan P, Hope Trant M, Laurie W. Non-Bancroftian Elephantiasis in Tanganyika. British Medical Journal 1956;1(4960):209-10.

45. de Lalla F, Zanoni P, Lunetta Q, Moltrasio G. Endemic non-filarial elephantiasis in Iringa District, Tanzania: a study of 30 patients. . Trans R Soc Trop Med Hyg 1988;82( 6):895-7.

46. Lowenthal LJA. On the probable inclusion of several diseases in the title "mossy" foot. Ann Trop Med Parasitol 1934;28:47-57.

47. Onapa AW, Simonsen PE, Pedersen EM. Non-filarial elephantiasis in the Mt. Elgon area (Kapchorwa District)of Uganda. Acta Trop. 2001;78(2):171-6.

48. Dwek P, Yuan KL, Wafer M, Cherniak W, Pace R, Malhamé I, et al. Case Report and Literature Review: Podoconiosis in Southwestern Uganda. International Journal of TROPICAL DISEASE & Health 2015;9(3):1-7.

49. Kihembo C, Masiira B, Lali WZ, Matwale GK, Matovu JKB, Kaharuza F, et al. Risk Factors for Podoconiosis: Kamwenge District, Western Uganda, September 2015. Am J Trop Med Hyg. 2017;96(6):1490-6.

50. Price EW, Bailey D. Environmental factors in the etiology of endemic elephantiasis of the lower legs in tropical Africa. . Trop Geogr Med 1984;36(1):1-5.

51. Sinha M, Saha AK, editors. A possible linkage of non-filarial elephantiasis “podoconiosis” with geochemical factors, Bhiwapur area, Nagpur district, Maharashtra. Proceedings of the workshop on Medical Geology IGCP-454; 2004 February 3–4 Nagpur, India.

52. Russel S, Rao CK, Rao CK. Prevalence of nonfilarial elephantiasis in selected towns in India. The Journal of communicable diseases. 1983;15(3):216-8. PubMed PMID: 6672090.

53. Kalra NL. Non-fllarial elephantiasis in Bikaner, Rajasthan. Journal of Communicable Diseases 1976;8 (4):337-40.

54. Tada MS, Marsden PD. Probable podoconiosis in Brasilia. Rev Soc Bras Med Trop. 1993;26(4):255.

55. Ruiz-Arnau R. Filariasis and Its Relation to Other Tropical Lymphopathies Am J Trop Med Hyg. 1922;s1-2(2):151-7.

56. De Meira MTV, Somoes TS, Nogueira JFP. On the existence of mossy foot on the island of St Nicolau (Cape Verde). Ann Inst Med Trop (Lisbon) 1947;4:269-79.

57. Dubois A, Forro M. Contribution B l’ktiologie de l’elephantiasis Congolais. Annales de la So&J Be& de M&&e Tropicale 1939;19(13).

58. Russel S, Krishna Rao C, Rao CK. Prevalence of nonfilarial elephantiasis in selected towns in India. The Journal of communicable diseases. 1983;15(3):216-8. PubMed PMID: 6672090.
